# Supplementary material for: Food Web Assembly Rules for Generalized Lotka-Volterra Equations
Source: PLoS Comput Biol. 2016 Feb 1;12(2):e1004727. doi: 10.1371/journal.pcbi.1004727 (PMC4734619; doi:10.1371/journal.pcbi.1004727)
Supplement: S1 Text — Additional technical details on mathematics and data analysis. (PDF) [file pcbi.1004727.s001.pdf]

Food web assembly rules for generalized Lotka-Volterra equations.

(S1 Text: Supporting Information.)

Jan O. Haerter

email: haerter@nbi.dk

Namiko Mitarai

email: mitarai@nbi.dk

Kim Sneppen

email: sneppen@nbi.dk

Center for Models of Life, Niels Bohr Institute

University of Copenhagen

Blegdamsvej 17, DK-2100, Copenhagen, Denmark

January 13, 2016

Abstract

This supplementary information provides additional technical detail which may be interesting to specialist readers as well as introductory examples for non-specialists, but is not required for the understanding of the main text. We provide more detail on the derivation of our main equations (Sec. S1) and their steady state (Sec. S2). We give a detailed justification of the terms *resource-limited* and *consumer-limited* state as used in the main text (Sec. S3), a simple example demonstrating the coexistence between two consumers and two producers (Sec. S4), as well as a brief summary of the assembly rules (Sec. S5). We offer in-depth discussion of food webs containing omnivores (Sec. S6) and the case of food webs where basal species feed on a set of separate nutrients (Sec. S7). We also discuss a modification of the assembly rules for the case of the type-II functional response (Sec. S8). Details on the data analysis performed with empirical food webs, including a discussion on concomitant predation, are given in Sec. S9. Simulation results for a model food web are provided in Sec. S10.

Contents

S1 Food web ecology **4**

S2 Steady state and its stability **6**

S2.1 Steady state 6

S2.2 Stability 6

S2.2.1 When is Q semipositive definite? 10

S3 Resource versus consumer limitation **11**

S4 Species definition and example of two shared niches **14**

S5 Assembly rules for a food web with strict trophic levels **17**

S6 Omnivory and parasitism **18**

S6.1 Example of omnivory 19

S6.2 Generalist omnivory 20

S6.3 Pairing requirements for food-webs with omnivores 20

S7 Basal species with separate nutrients **23**

S8 Species link requirement to include predator saturation **24**

S8.1 Model equation with type-II response 24

S8.2 Constraints from steady state condition and extended species pairing 25

S8.2.1 Top predators 25

S8.2.2 Intermediate species 25

S8.2.3 Basal species 27

S8.2.4 Summary of the extended species pairing requirement 27

S9 Data Analysis **29**

S9.1 Data considerations 29

S9.1.1 Food web data 29

S9.1.2 Technical considerations on parasites 29

S9.1.3 Matrix rank, basal species and nutrient sources 30

S9.1.4 Treatment of detritus and particulate organic matter (POM) in the data 30
S9.1.5 Approximate trophic levels 30
S9.2 Analysis of food web matrices of free-living species 31
S9.2.1 Trophic levels and omnivory 31
S9.2.2 Trophic structure of food web matrices 32
S9.2.3 Discussion of individual matrices (details) 33
S9.3 Analysis of food web matrices including parasite species 34
S9.3.1 Trophic positions with parasites 34
S9.3.2 Discussion of individual food webs (details) 35
S9.3.3 Concomitant predation 36
S9.4 Removal of species from food web 39

S10 Modeling 42

S10.1 Idealized food web structure 42
S10.2 Simulations of omnivore addition within the free-living web 44
S10.3 Simulations of parasite addition 44
S10.4 Simulations of the effect of concomitant predation 48

S1 Food web ecology

To model food web ecology, we employ the widely-used, generalized Lotka-Volterra equations (MacArthur, 1967) which assume a well-mixed system, i.e. a system where spatial fluctuations are negligible. The links in a food web are descriptions of predominantly predator-prey relationships between species on neighboring trophic levels (Montoya et al., 2006) but could also constitute mutualist interactions (Bascompte et al., 2005).

The lowest trophic level of the food chain, primary producers such as plants or microbes, draw energy from the sun and elementary chemical compounds. We define a distinct species as an organism of distinguishable phenotype. We first separate species with regard to their trophic levels and direct interactions between species occur only between those on neighboring trophic levels. To model the food chain, we require two different types of equations. For n_1 primary producers with (population or mass) densities $S_i^{(1)}$, $i = 1, \ldots, n_1$, residing at the first trophic level, the general form of the time-evolution equations is (MacArthur, 1967; Gross et al., 2009)

$$\frac{1}{S_i^{(1)}} \frac{dS_i^{(1)}}{dt} = k_i^{(1)} \left(1 - \sum_{j=1}^{n_1} p_{ji} S_j^{(1)} \right) - \alpha_i^{(1)} - \sum_{k=1}^{n_2} \eta_{ki}^{(2,1)} S_k^{(2)} \ . \tag{S1}$$

In Eq. S1, competition between the primary producers for basic resources is described by a normalized overall carrying capacity in the first term on the RHS (Smith and Slatkin, 1973). $k_i^{(1)}$ ($\alpha_i^{(1)}$) denote the growth (decay) rates of $S_i^{(1)}$, p_{ji} describe differential consumption of the basic resources by the $S_i^{(1)}$ and $\eta_{ki}^{(2,1)}$ are the interaction coefficients between a species $S_k^{(2)}$ at the second with species $S_i^{(1)}$ at the first trophic level. For positive $\eta_{ki}^{(2,1)}$ the consumer $S_k^{(2)}$ yields a net increase of decay rate of basal species $S_i^{(1)}$. For negative $\eta_{ki}^{(2,1)}$ (weak mutualism), a positive effect on the growth rate of $S_i^{(1)}$ results. Primary producers compete for resources when $p_{ji} > 0$ for $i \neq j$, but the equation also allows for the description of the case of direct competition, cooperation, as well as growth on non-overlapping resources ($p_{ji} = \delta_{ji}$).

For species $S_k^{(l)}$ at any trophic level $l > 1$ we write

$$\frac{1}{S_k^{(l)}} \frac{dS_k^{(l)}}{dt} = \sum_{m=1}^{n_{l-1}} \beta_{km}^{l,l-1} \cdot \eta_{km}^{l,l-1} \cdot S_m^{(l-1)} - \sum_{p=1}^{n_{l+1}} \eta_{pk}^{l+1,l} \cdot S_p^{(l+1)} - \alpha_k^{(l)} \ , \tag{S2}$$

where $\beta_{km}^{l,l-1}$ are the efficiencies of reproduction of species $S_k^{(l)}$ when consuming species $S_m^{(l-1)}$ and the other coefficients are defined analogously to those in Eq. S1. For top predators, residing on the L'th trophic level, there are no enemies, hence the coefficients $\eta_{pk}^{L+1,L}$ will vanish. Recycling of the top predators' biomass will then only occur through their natural death rates $\alpha_k^{(L)}$. For a species' phenotype to be distinguishable, it must be characterized by a unique combination of the parameters. In the above equations, all parameters and observables have been made dimensionless by normalizing population

densities to the primary producer carrying capacity, growth and decay rates to the maximal primary producer growth rate, and interaction rates to the ratio of maximal growth rate and carrying capacity. Note that Eqs S1 and S2 differ by the respective consumption of resources: Primary producers consume supplies of physical or chemical energy which are replenished at a constant flux. Species at any higher trophic level depend on the availability of prey at the neighboring trophic level below.

S2 Steady state and its stability

The ecological dynamics are taken to occur without rapid evolution, i.e. we are considering systems where species richness is not a consequence of transient rise and decay of species. In the following, we describe how to obtain the interaction matrix $\mathcal{R}$ in the steady-state equations (Sec. S2.1). We then discuss stable networks for any combination of species richnesses that is in accordance with Eq. 6, main text (Sec. S2.2).

S2.1 Steady state

To describe sustainable food webs, we demand the time-derivatives on the LHS of Eqs S1 and S2 to vanish and arrive at a set of equations that are linear in the densities of any of the species:

$$\sum_{j=1}^{n_1} p_{ji} S_j^{(1)} + \sum_{k=1}^{n_2} \frac{\eta_{ki}^{(2,1)}}{k_i^{(1)}} S_k^{(2)} = \frac{k_i^{(1)} - \alpha_i^{(1)}}{k_i^{(1)}} \equiv \tilde{k}_i^{(1)} \tag{S3}$$

$$\sum_{m=1}^{n_{l-1}} \beta_{km}^{(l,l-1)} \eta_{km}^{(l,l-1)} S_m^{(l-1)} - \sum_{p=1}^{n_{l+1}} \eta_{pk}^{(l+1,l)} S_p^{(l+1)} \equiv \alpha_k^{(l)} . \tag{S4}$$

Collecting all constant coefficient (LHS of Eqs S3 and S4) in the vector $\mathbf{k}$ and all interaction coefficients on the RHS in the interaction matrix $\mathcal{R}$, we have the linear matrix equation $\mathcal{R} \cdot \mathbf{S} = \mathbf{k}$, where $\mathbf{S}$ is the vector of all species densities. For completely shared nutrients, the competition factors $p_{ji} = 1$. In the opposite extreme, where each basal species draws energy from a distinct nutrient source, $p_{ji} = \delta_{ji}$ with δ_{ji} the Kronecker delta function. When re-organizing the terms in the sub-matrix of interactions between species on the basal trophic level, the square matrix $\mathcal{A}_1$ is obtained by subtracting rows 2 to n_1 from row 1. After this linear transformation we yield the modified matrix $\mathcal{R}'$, shown in Fig. 1a (main text). Apart from the first row, $\mathcal{R}'$ has a block structure with nonzero entries only for interactions between neighboring trophic levels (Fig. 1a, main text). Steady-state solutions are then given by $\mathbf{S}^* \equiv \mathcal{R}^{-1}\mathbf{k}$, thus a unique solution $\mathbf{S}^*$ requires that $\mathcal{R}$ be invertible, hence $det(\mathcal{R}) \neq 0$ (Otto and Day, 2007). In the following we first consider the case of shared nutrients ($p_{ji} = 1$) but in Sec. S7 also separate resources are discussed.

S2.2 Stability

To demonstrate conditions for stability, in the following we show how a Lyapunov function can be constructed, in analogy to Goh (1977) and Hofbauer and Sigmund (1988). Let us consider the generalized Lotka-Volterra equation (Eqs S1 and S2)

$$\dot{S}_i^{(l)} = w_i^{(l)} S_i^{(l)} \tag{S5}$$

with

$$w_i^{(1)} = k_i^{(1)} \left(1 - \sum_{j=1}^{n_1} p_{ji} S_j^{(1)} \right) - \alpha_i^{(1)} - \sum_{k=1}^{n_2} \eta_{ki}^{(2,1)} S_k^{(2)} \tag{S6}$$

for primary producers and

$$w_k^{(l)} = \sum_{m=1}^{n_{l-1}} \beta_{km}^{l,l-1} \cdot \eta_{km}^{l,l-1} \cdot S_m^{(l-1)} - \sum_{p=1}^{n_{l+1}} \eta_{pk}^{l+1,l} \cdot S_p^{(l+1)} - \alpha_k^{(l)} \tag{S7}$$

for higher levels $l > 1$. For the top level L, the last term in the right hand side will be zero. Let us now assume that a feasible solution exists. For clarity and ease of notation, in the following derivation we use the symbol $f_i^{(l)}$ wherever the steady-state density of a species i at a trophic level l is meant (the symbol $\mathbf{S}^*$ used in the main text is the vector of all steady-state densities $f_i^{(l)}$). The population for a given feasible solution $f_i^{(l)}$ satisfies

$$0 = k_i^{(1)} \left(1 - \sum_{j=1}^{n_1} p_{ji} f_j^{(1)} \right) - \alpha_i^{(1)} - \sum_{k=1}^{n_2} \eta_{ki}^{(2,1)} f_k^{(2)} \tag{S8}$$

$$\Leftrightarrow -k_i^{(1)} + \alpha_i^{(1)} = - \sum_{j=1}^{n_1} k_i^{(1)} p_{ji} f_j^{(1)} - \sum_{k=1}^{n_2} \eta_{ki}^{(2,1)} f_k^{(2)} \tag{S9}$$

and

$$0 = \sum_{m=1}^{n_{l-1}} \beta_{km}^{l,l-1} \cdot \eta_{km}^{l,l-1} \cdot f_m^{(l-1)} - \sum_{p=1}^{n_{l+1}} \eta_{pk}^{l+1,l} \cdot f_p^{(l+1)} - \alpha_k^{(l)} \tag{S10}$$

$$\Leftrightarrow \alpha_k^{(l)} = \sum_{m=1}^{n_{l-1}} \beta_{km}^{l,l-1} \cdot \eta_{km}^{l,l-1} \cdot f_m^{(l-1)} - \sum_{p=1}^{n_{l+1}} \eta_{pk}^{l+1,l} \cdot f_p^{(l+1)}. \tag{S11}$$

Replacing $k_i^{(1)} - \alpha_i^{(1)}$ and $\alpha_i^{(l)}$ in the expressions for w's, we have

$$w_i^{(1)} = - \sum_{j=1}^{n_1} k_i^{(1)} p_{ji} \left(S_j^{(1)} - f_j^{(1)} \right) - \sum_{k=1}^{n_2} \eta_{ki}^{(2,1)} \left(S_k^{(2)} - f_k^{(2)} \right) \tag{S12}$$

and

$$w_k^{(l)} = \sum_{m=1}^{n_{l-1}} \beta_{km}^{l,l-1} \cdot \eta_{km}^{l,l-1} \cdot \left(S_m^{(l-1)} - f_m^{(l-1)} \right) - \sum_{p=1}^{n_{l+1}} \eta_{pk}^{l+1,l} \cdot \left(S_p^{(l+1)} - f_p^{(l+1)} \right). \tag{S13}$$

Now, let us define a function

$$V(S) = \sum_{l=1}^{N} \sum_i c_i^{(l)} \left(S_i^{(l)} - f_i^{(l)} \ln S_i^{(l)} \right) \tag{S14}$$

with constants $c_i^{(l)}$, which we will determine later. We have

$$\dot{V}(S) = \sum_{l=1}^{N}\sum_{i} c_i^{(l)} \left(\dot{S}_i^{(l)} - f_i^{(l)} \frac{\dot{S}_i^{(l)}}{S_i^{(l)}} \right) \tag{S15}$$

$$= \sum_{l=1}^{N}\sum_{i} c_i^{(l)} \left(S_i^{(l)} - f_i^{(l)} \right) w_i^{(l)}. \tag{S16}$$

Using the expressions for $w_i^{(l)}$, we get

$$\begin{aligned}
\dot{V}(S) &= \sum_i c_i^{(1)} \left(S_i^{(1)} - f_i^{(1)} \right) \left[-\sum_{j=1}^{n_1} k_i^{(1)} p_{ji} \left(S_j^{(1)} - f_j^{(1)} \right) - \sum_{k=1}^{n_2} \eta_{ki}^{(2,1)} \left(S_k^{(2)} - f_k^{(2)} \right) \right] \\
&\quad + \sum_{l=2}^{N-1} \sum_k c_k^{(l)} \left(S_k^{(l)} - f_k^{(l)} \right) \left[\sum_{m=1}^{n_{l-1}} \beta_{km}^{l,l-1} \cdot \eta_{km}^{l,l-1} \cdot \left(S_m^{(l-1)} - f_m^{(l-1)} \right) \right. \\
&\quad \left. - \sum_{p=1}^{n_{l+1}} \eta_{pk}^{l+1,l} \cdot \left(S_p^{(l+1)} - f_p^{(l+1)} \right) \right] \\
&\quad + \sum_k c_k^{(N)} \left(S_k^{(N)} - f_k^{(N)} \right) \left[\sum_{m=1}^{n_{l-1}} \beta_{km}^{l,l-1} \cdot \eta_{km}^{l,l-1} \cdot \left(S_m^{(l-1)} - f_m^{(l-1)} \right) \right] \\
&= -\sum_i^{n_1} \sum_{j=1}^{n_1} c_i^{(1)} k_i^{(1)} p_{ji} \left(S_i^{(1)} - f_i^{(1)} \right) \left(S_j^{(1)} - f_j^{(1)} \right) \\
&\quad - \sum_{l=2}^{N} \sum_{i=1}^{n_{l-1}} \sum_{k=1}^{n_l} \left[c_i^{(l-1)} \eta_{ki}^{(l,l-1)} - c_k^{(l)} \beta_{ki}^{l,l-1} \eta_{ki}^{l,l-1} \right] \left(S_i^{(1-1)} - f_i^{(1-1)} \right) \left(S_k^{(l)} - f_k^{(l)} \right)
\end{aligned} \tag{S17}$$

If we can choose the values of the c's such that the *multiplication rule*

$$c_i^{(l-1)} = c_k^{(l)} \beta_{ki}^{l,l-1} \tag{S18}$$

is fulfilled, the system is globally stable. Noticeably, this multiplication rule does not involve any of the coefficients $\eta_{ki}^{(l,l')}$, the link strengths can hence still be modified, or links removed, by adjustments to these coefficients.

If the multiplication rule (Eq. S18) is satisfied, the expression Eq. S17 becomes extremely simple. In particular, satisfying Eq. S18 is possible at least in the following cases.

1. When the network structure is a tree there is only one path between any pair of nodes. In this case, there is a unique link from a node at the level l to a node at the level $l-1$. Therefore it is possible to find the values $c_k^{(l)} = c_i^{(l-1)}/\beta_{ki}^{l,l-1}$ for $l \geq 2$.

2. If the network structure is not a tree, this can be satisfied only if $\beta_{ki}^{l,l-1}$ satisfies $\beta_{ki}^{l,l-1} = c_k^{(l)}/c_i^{(l-1)}$. This is possible for any network structure.

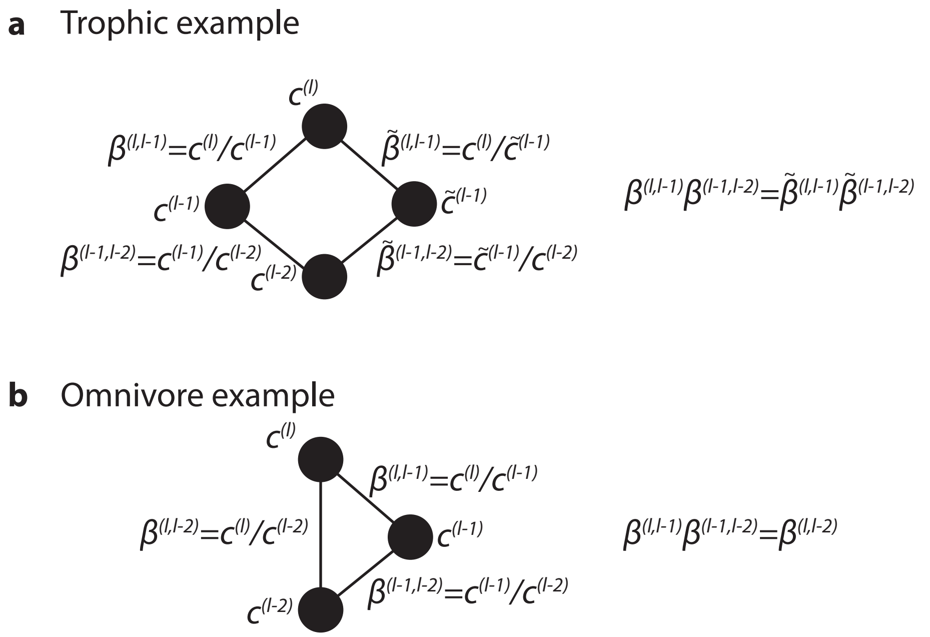

Figure A: **Schematic showing two examples of loops.** **a**, A loop is formed by two paths connecting a species at level l and a species at level $l-2$. **b**, The species at level l is an omnivore and consumes a species both at level $l-1$ and $l-2$.

For the case 2, we comment on the existence of loops, i.e. structures where two different paths connect two species (Fig. A). The rule (Eq. S18) can be fulfilled if and only if the products of β's along any different paths between the two given species are equal (Fig. A). Notably, this encompasses the loops involved in food webs with strict trophic levels (Fig. Aa) and those involving omnivores (Fig. Ab). Although our formalism above only considered interactions between trophic levels l and $l-1$, there is nothing that prevents us from extending this formalism to interactions with levels further away, thereby including omnivory. Hence, given that a steady state exists, stability is possible by proper parameter choice for systems involving loops.

In either of these cases 1 and 2, the value of $c_i^{(1)}$ is not determined. We then have

$$\dot{V}(S) = -\sum_{i}^{n_1}\sum_{j=1}^{n_1} c_i^{(1)} k_i^{(1)} p_{ji} \left(S_i^{(1)} - f_i^{(1)}\right)\left(S_j^{(1)} - f_j^{(1)}\right). \tag{S19}$$

We can define a real symmetric matrix $Q_{ij} = \frac{1}{2}\left(c_i^{(1)} k_i^{(1)} p_{ji} + c_j^{(1)} k_j^{(1)} p_{ij}\right)$ to rewrite this as

$$\dot{V}(S) = -\sum_{i}^{n_1}\sum_{j=1}^{n_1} Q_{ij} \left(S_i^{(1)} - f_i^{(1)}\right)\left(S_j^{(1)} - f_j^{(1)}\right). \tag{S20}$$

If the $n_1 \times n_1$ real symmetric matrix Q is semipostitive definite [1], we have a Lyapunov function $V(S)$ that satisfies $\dot{V}(S) \leq 0$.

[1] A real symmetric matrix is semipositive definite if and only if all its eigenvalues are positive or zero. In this case, there is a real matrix P that makes PQP^{-1} to be diagonal matrix with Q's eigenvalues and $P^{-1} = P^t$. Since $\sum_{i,j} x_i Q_{ij} x_j = \vec{x}^t Q \vec{x} = \vec{x}^t Q \vec{x} = (P\vec{x})^t (PQP^{-1}) P\vec{x}$, $\sum_{i,j} x_i Q_{ij} x_j = \sum_{i=1}^{n_1} \lambda_i y_i^2 \geq 0$ if all $\lambda_i \geq 0$, with $\vec{y} = P\vec{x}$.

S2.2.1 When is Q semipositive definite?

The following two cases are easy to consider.

When $p_{ij} = \delta_{ij}$. In this case, Q is semipositive definite as long as all $c_i^{(1)} k_i^{(1)}$ are positive. If for example we set $c_i^{(1)} = 1$, then Eq. S21 becomes

$$\dot{V}(S) = -\sum_i^{n_1} k_i \left(S_i^{(1)} - f_i^{(1)} \right)^2 \leq 0. \tag{S21}$$

When $p_{ij} = 1$. In this case, Q is semipositve definite if $c_i^{(1)} = 1/k_i^{(1)}$. In this case $Q_{ij} = 1$ and the eigenvalues of Q are n_1 and zero. Eigenvector for the eigenvalue n_1 is the vector filled with ones. In this case, Eq. S21 will become

$$\dot{V}(S) = -\left(\sum_i^{n_1} \left(S_i^{(1)} - f_i^{(1)} \right) \right)^2 \leq 0\ . \tag{S22}$$

It is straightforward to extend this to the case where p_{ij} is block diagonal with blocks with ones and blocks with delta-functions.

S3 Resource versus consumer limitation

Trophic cascades are changes in the population densities of consumers and resources in food chains when a top predator is added or removed, but can also be triggered by changes in nutrient availability (Heath et al., 2014). Exemplified by the growth of a simple food chain — starting with a single species — we discuss the alternating concentration of biomass on odd, respectively even, trophic levels.

A freely growing basal species, $S_1^{(1)}$ according to Eq. S3 will reach the steady state population density $S_1^{(1)} = 1 - \alpha_1^{(1)}/k_1^{(1)}$ and can reach the system carrying capacity (here set to unity) by increasing its growth rate $k_1^{(1)}$ relative to its decay rate $\alpha_1^{(1)}$. Consider now a simple ecosystem consisting of only two trophic levels with a single species on each level, i.e. a basal and a consumer species (Fig. B). Eqs S3 and S4 then become

$$1 - \frac{\alpha_1^{(1)}}{k_1^{(1)}} = S_1^{(1)} + \frac{\eta_{11}^{(2,1)}}{k_1^{(1)}} S_1^{(2)} \tag{S23}$$

$$\alpha_1^{(2)} = \beta_{11}^{(2,1)} \eta_{11}^{(2,1)} S_1^{(1)} \, . \tag{S24}$$

Hence, the basal species population density

$$S_1^{(1)} = \frac{\alpha_1^{(2)}}{\beta_{11}^{(2,1)} \eta_{11}^{(2,1)}} \tag{S25}$$

is entirely determined by the trophic interaction and the decay rate of its consumer and $S_1^{(1)}$ will not primarily be limited by the carrying capacity of the system. In its simplest form this exemplifies what is meant by the transition from the resource to the consumer-limited state.

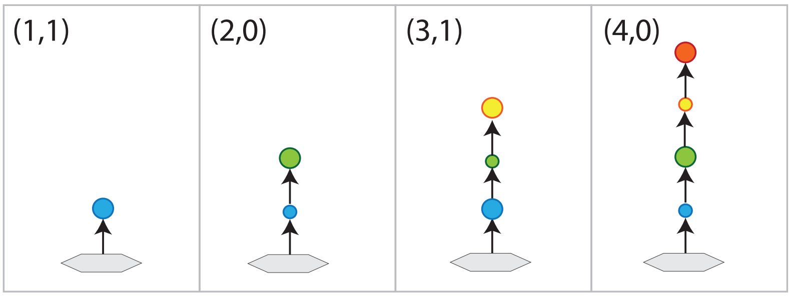

Figure B: **Simple food web.** Food web containing only a single species at each trophic level. Schematic shows re-distribution of biomass upon addition of trophic levels. Large (small) circles indicate population with large (small) biomass. Labels show the total number of species and the difference $N_o - N_e$.

Continuing further, we consider a single species $S_1^{(3)}$ on the third trophic level and obtain the solution

$$\begin{aligned} 1-\frac{\alpha_1^{(1)}}{k_1^{(1)}} &= S_1^{(1)}+\frac{\eta_{11}^{(2,1)}}{k_1^{(1)}}S_1^{(2)} && \text{(S26)}\\ \alpha_1^{(2)} &= \beta_{11}^{(2,1)}\eta_{11}^{(2,1)}S_1^{(1)}-\eta_{11}^{(3,2)}S_1^{(3)} && \text{(S27)}\\ \alpha_1^{(3)} &= \beta_{11}^{(3,2)}\eta_{11}^{(3,2)}S_1^{(2)}\;. && \text{(S28)} \end{aligned}$$

Hence, $S_1^{(2)}=\alpha_1^{(3)}/\beta_{11}^{(3,2)}\eta_{11}^{(3,2)}$ is now entirely specified by the interaction with $S_1^{(3)}$ and the decay rate $\alpha_1^{(3)}$. In turn, the basal species density

$$S_1^{(1)}=1-\frac{\alpha_1^{(1)}}{k_1^{(1)}}\left(1+\frac{\alpha_1^{(3)}}{\alpha_1^{(1)}}\frac{\eta_{11}^{(2,1)}}{\eta_{11}^{(3,2)}}\frac{1}{\beta_{11}^{(3,2)}}\right) \tag{S29}$$

again can approach the carrying capacity by increasing its growth rate relative the the decay rate. Note that Eq. S29 describes a reduction of the producer species both due to its own death rate and the death of its indirect support on level 3.

A similar analysis of the 4-level system reveals that in that case the population of the producer increases from its previous level (Eq. S25) to a larger level because its predator (the species at level 2) has to "carry" the death of its support (level 4), i.e.

$$S_1^{(1)}=\frac{1}{\beta_{11}^{(2,1)}\eta_{11}^{(2,1)}}\left(\alpha_1^{(2)}+\frac{\eta_{11}^{(3,2)}}{\eta_{11}^{(4,3)}}\frac{1}{\beta_{11}^{(4,3)}}\alpha_1^{(4)}\right)\;. \tag{S30}$$

It is easy to continue the sequence by adding trophic levels with single species and the alternation between resource and consumer limit will be obeyed. More generally, with species diversities $\{n_1,\ldots,n_L\}$ on the L trophic levels, in the consumer-limited case, the decay rates for consumers and their respective interactions with producer species will set the population densities of the $N_o=N_e$ resource species. In the resource limited state, one degree of freedom for basal species will be preserved. In principle, sustainable networks could then be formed where one basal species again approaches the system carrying capacity by optimizing its own growth and decay rates, i. e. this basal species could be freed from exploitation by consumers. Conversely, in a sustainable consumer-limited state, all species belonging to the resource support *must* be exploited by consumers. None of the resource species can — even in principle — be freed.

This can be made more explicit: Consider a system of only two trophic levels with diversities n_1 and n_2. If $n_2=n_1$, the n_2 equations (S4) define n_2 weighted sums of the $n_1=n_2$ species at trophic level 1. It is then always possible to yield an expression for the sum $S^{(1)}\equiv\sum_{m=1}^{n_1}S_m^{(1)}$ of total population density on the basal trophic level which depends only on the decay rates $\alpha_k^{(l)}$ of and interaction coefficients with species on the second trophic level. Hence, the population density $S^{(1)}$ will not depend on the growth or decay rates of species on the basal level. In other words, species fitness does not improve by increasing

its growth rate, but remains solely governed by the species' predators (Thingstad, 2000).

Conversely, if $n_2 = n_1 - 1$, only the sum of $n_1 - 1$ species on level 1 can be expressed in terms of the respective decay and interaction coefficients. The remaining degree of freedom will then be fixed by Eq. S3 where the carrying capacity will be felt by the basal species. Generalizing to L trophic levels, $n_2 - n_1$ is the number of degrees of freedom on level 1 controllable by level 2. However, as n_3 constraints can be imposed by level 3 onto level 2, the net limitation is then $n_2 - n_1 - n_3$. This sequence will continue analogously until n_L is reached and we are left with the familiar conditions $N_o - N_e \in \{0, 1\}$, a difference that defines the number of degrees of freedom remaining for the basal level.

S4 Species definition and example of two shared niches

A trophic species is defined as all species sharing a set of predators and prey. An example is shown in Fig. C.a and b. The strength of the links connecting the respective consumers and resources is thereby not relevant, as long as the link exists. I.e., one consumer may predominantly feed on one prey while the other consumer predominantly feeds on the other prey (Fig. Cc).

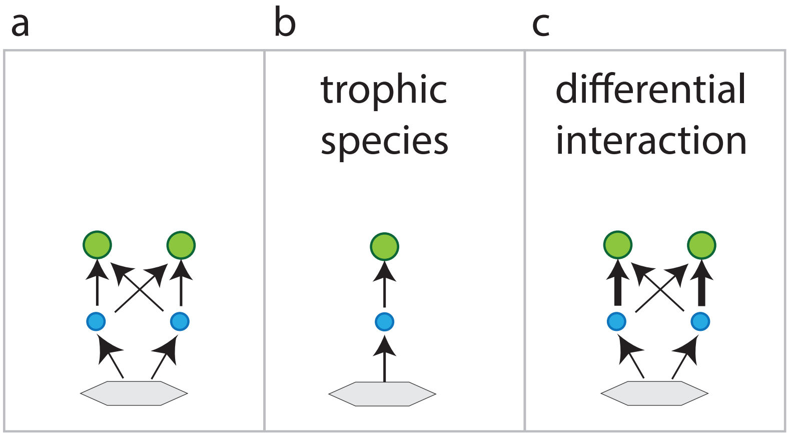

Figure C: **Food web with two niches.** **a**, Example of food web with two basal and two consumer species. Basal species share nutrients and consumer species and can be combined to a single trophic species. Consumer species share resources and can also be combined to a single trophic species. **b**, Food web with species aggregated into trophic species. **c**, As in (a) but showing differential predation rates.

In our approach, we also distinguish species by the interaction strength with other species. For example, when two species feed on the same two prey species, but they do so at differential rates, we will still consider them distinguishable species. The reasoning behind this is twofold: First, for the competitive exclusion principle to be relevant, we need to be able to discuss "perfect" competitors, i.e. those that share a resource. Aggregating the two consumers into a single trophic species would immediately remove this situation, the discussion of competitive exclusion would hence be meaningless from the outset. Second, to discuss how the competitive exclusion principle can be generalized, we do need to discuss situations where species, despite sharing prey and predator species, may still coexist. This is at the core of our discussion.

In the specific case of Fig. C.a, by allowing complementary interaction rates, the two species occupy distinct niches, even though they principally share both prey species. Coexistence between two competing species can be rationalized as a form of trade-off (Tilman, 1990), where one species may be better at preying on one resource than the other but thereby inevitably sacrificing efficiency in preying on the second resource species. To make this more explicit, we now discuss in detail the example shown in (Fig. C.c). In terms of its steady state condition:

$$S_1^{(1)} + S_2^{(1)} + \frac{\eta_{11}^{(2,1)}}{k_1^{(1)}} S_1^{(2)} + \frac{\eta_{21}^{(2,1)}}{k_1^{(1)}} S_2^{(2)} = \tilde{k}_1^{(1)} \quad \text{(S31)}$$

$$S_1^{(1)} + S_2^{(1)} + \frac{\eta_{12}^{(2,1)}}{k_2^{(1)}} S_1^{(2)} + \frac{\eta_{22}^{(2,1)}}{k_2^{(1)}} S_2^{(2)} = \tilde{k}_2^{(1)} \quad \text{(S32)}$$

$$\beta_{11}^{(2,1)} \eta_{11}^{(2,1)} S_1^{(1)} + \beta_{12}^{(2,1)} \eta_{12}^{(2,1)} S_2^{(1)} = \alpha_1^{(2)} \quad \text{(S33)}$$

$$\beta_{21}^{(2,1)} \eta_{21}^{(2,1)} S_1^{(1)} + \beta_{22}^{(2,1)} \eta_{22}^{(2,1)} S_2^{(1)} = \alpha_2^{(2)} \quad \text{(S34)}$$

To simplify the discussion, we now set all $\beta_{ij}^{(2,1)} \equiv \beta$, $\alpha_i^{(l)} \equiv \alpha$ and ask only for symmetric solutions where $\eta_{12}^{(2,1)} = \eta_{21}^{(2,1)} \equiv \eta_\times$ and $\eta_{11}^{(2,1)} = \eta_{22}^{(2,1)} \equiv \eta_{||}$. Note that we have also dropped the superscripts in these coefficients. Using these simplifications, we arrive at solutions

$$\begin{aligned} S_1^{(1)} = S_2^{(1)} &= \frac{\alpha}{\beta} \frac{1}{\eta_{||} + \eta_\times} \qquad \text{(S35)} \\ S_1^{(2)} &= \frac{1}{\eta_{||}^2 - \eta_\times^2} \left(\eta_{||}(k_1 - \alpha) - \eta_\times (k_2 - \alpha) + 2\frac{\alpha}{\beta} \left(\frac{\eta_\times}{\eta_{||} + \eta_\times} k_2 - \frac{\eta_{||}}{\eta_{||} + \eta_\times} k_1 \right) \right) \\ S_2^{(2)} &= \frac{1}{\eta_{||}^2 - \eta_\times^2} \left(\eta_{||}(k_2 - \alpha) - \eta_\times (k_1 - \alpha) + 2\frac{\alpha}{\beta} \left(\frac{\eta_\times}{\eta_{||} + \eta_\times} k_1 - \frac{\eta_{||}}{\eta_{||} + \eta_\times} k_2 \right) \right) , \end{aligned}$$

where the first equation should be compared to Eq. S25. From these solutions we see that the basal species are now completely dependent on the interaction rates with the consumer species. $S_1^{(1)}$ and $S_2^{(1)}$ become inversely proportional to the efficiency of predation, β, and inversely proportional to the sum of contact rates with the predatory species. From the densities of the consumer species, $S_1^{(2)}$ and $S_2^{(2)}$ we see that they become singular as the difference between (squared) link strengths vanishes, i.e. $\eta_{||} - \eta_\times \to 0$. This corresponds to the case of the competitive exclusion principle, when generalizing to a niche consisting of two basal species. Equal link strength is equivalent to perfect competition between the two consumer species. The singularity could only be lifted by choosing $k_1 = k_2$, a combined situation where we would consider the basal species to be identical.

Conversely, solutions do exist when link strengths are not equal, e.g. $\eta_{||} \gg \eta\times$. In that case, feasible solutions imply inequalities between the parameters, e.g.

$$\alpha^{-1} > k_1^{-1} + (\eta_{||}\beta/2)^{-1} \ , \ \text{respectively} \ \alpha^{-1} > k_2^{-1} + (\eta_{||}\beta/2)^{-1} \ . \quad \text{(S36)}$$

This is to say that the "free survival time“, α^{-1} must be sufficiently long to outweigh the production time $k_{1/2}^{-1}$ and effective "processing time“ $(\eta_{||}\beta/2)^{-1}$. Solutions with a component resulting from $\eta_\times$ would yield contributions to the consumers densities from both basal species (compare Eq. S36).

The above example, with each consumer species developing a weak and a strong link to a resource species, could in practice be generated by functional relations between interaction parameters, known as

trade-offs Tilman (1990).

Infinite solutions: We return to Eqs S33 and S34. Consider the case where

$$\frac{\beta_{11}^{(2,1)}\eta_{11}^{(2,1)}}{\beta_{21}^{(2,1)}\eta_{21}^{(2,1)}} = \frac{\beta_{12}^{(2,1)}\eta_{12}^{(2,1)}}{\beta_{22}^{(2,1)}\eta_{22}^{(2,1)}} = \frac{\alpha_1^{(2)}}{\alpha_2^{(2)}} \ . \tag{S37}$$

This choice of parameters would make Eqs S33 and S34 equivalent and thus allow infinite solutions for $S_1^{(1)}$ and $S_2^{(1)}$. While mathematically possible, complete equivalence of parameters is biologically exceedingly unlikely. In natural systems, even a small imbalance in the magnitude of parameters would allow one of the species to become dominant. Further, it has previously been shown that the case of very similar parameter values leads to very small probabilities of coexistence (Haerter and Sneppen, 2012), simply because the population redistribution between the two species would perform a random walk, until one is exposed to the absorbing state of zero population.

S5 Assembly rules for a food web with strict trophic levels

The assembly rules state the minimal conditions for a sustainable steady state of a food web, which in turn requires that the determinant of its interaction matrix $\mathcal{R}$ is nonzero. Computationally, this can be examined by assigning random values for all nonzero entries, and subsequently calculating the rank of $\mathcal{R}$. If the rank r is equal to the total number of species S, then the known interactions are sufficient to explain the sustainability of the food web. If the rank is less than S, the rank deficiency $d \equiv S - r$ measures a minimal bound for the number of additional links needed to sustain the food web.

The determinant-equal-zero rule can be reformulated in terms of the network structure. We here consider the case of fully bi-directional networks without species with varying food chain lengths, such as omnivores (i.e. all species must have strict trophic levels). The assembly rules can then be summarized as:

1) Food webs without omnivores require perfect matching to be sustainable. Perfect matching is the ability to cover a non-directed network with non-overlapping pairings (Lovász and Plummer, 1986). The determinant for symmetric networks can only be nonzero if there is perfect matching. In a food web, perfect matching is said to be obtained when all species participate in pairings, and each nutrient source can, but need not, participate in a pairing. The perfect matching rule will later be generalized when we consider omnivores or parasites (Sec. S6.3) and their possible concomitant links (Sec. S9.3.3).

2) The assembly rules imply general constraints for sustainability of purely trophic food webs:

$$\sum_{l=1}^{k} n_l(-1)^{k+l+1} \begin{cases} \leq (-1)^k & \forall \quad k \in [1, L]\,, \text{ if } N_o - N_e = 1 \\ \leq 0 & \forall \quad k \in [1, L]\,, \text{ if } N_o - N_e = 0 \end{cases} \tag{S38}$$

$$\text{and} \quad N_o - N_e \in \{0, 1\}\ , \tag{S39}$$

for the case of one common resource. Here N_o and N_e are again defined as the total species richness at all odd, respectively even, trophic levels. Eq. S38 does justice to the constraints imposed by the species richness at distinct trophic levels, as discussed in the main text. Violation of these constraints can e.g. by quantified by the difference $N_e - N_o$, which expresses to which extent the food web is in the consumer-dominated state. $N_e - N_o$ can be used to estimate the minimal rank deficiency of a given set of data for a food web with sharp trophic levels and an excess number of consumers. [2]

[2]Note: For the case of excess resource species, minimal rank deficiency could analogously be expressed by the term $N_o - N_e - \Delta$, with Δ chosen as the positive integer less than or equal to the number of distinct nutrient sources n_S, so that the term is minimized.

S6 Omnivory and parasitism

Omnivores (Fagan, 1997; Thompson et al., 2007) or some parasites (McCann, 2000; Bascompte et al., 2005; Lafferty et al., 2008; Dunne et al., 2013) or pathogens (Mordecai, 2011) that prey on multiple trophic levels, have been suggested to play a moderating role in food webs. When disregarding concomitant links (Secs S9.3.3, S10.4), parasites, despite their complex life-cycles (Huxham et al., 1996), can be seen to formally act as omnivores in our steady-state equations, when considering a given parasite species with its population as a whole. In our analysis, even the sign of interaction would not matter, i.e. also mutualistic interactions with species at distinct trophic levels, or a combination of different interaction types (e.g. antagonistic towards one species at one trophic level and mutualistic to another species at a distinct level) would be possible.

In terms of the assembly rules, omnivores whose prey includes species on a given trophic level l can be interpreted as trophic species on the level $l+1$. Specifically, consider an omnivore preying on all species at the L_g trophic levels $\{i_1, i_2, \ldots, i_{L_g}\}$. This omnivore can then be interpreted as a trophic consumer residing on any of the trophic levels $\{i_1+1, i_2+1, \ldots, i_{L_g}+1\}$. Consider now the introduction of this omnivore into an existing L-level food web containing only nearest-level trophic interaction, i.e. omnivores are absent prior to the addition. The L diversities are then given by $\{n_1, \ldots, n_L\}$. The result of introducing the omnivore is, that the assembly rules now allow the redefinition of any of the existing diversities at the corresponding trophic levels, i.e. n_{i_1+1} can now be redefined to become

$$\tilde{n}_{i^*+1} \equiv n_{i^*+1} + 1 , \tag{S40}$$

where $i^* \in \{i_1, i_2, \ldots, i_{L_g}\}$. Note that for any single omnivore this replacement can only be made for a single selection of the L_g trophic diversities $\{i_1, i_2, \ldots, i_{L_g}\}$. This means, once the omnivore has been associated with a given trophic level, it can no longer be re-interpreted as adding to species richness at another. That said, the previous assembly rules now still hold when the diversities n_i are replaced by diversities $\tilde{n}_i$, for $i \in \{1, \ldots, L\}$. Consider the introduction of an omnivore into an existing food web, which previously obeyed the assembly rules.

Using the guideline defined by Eq. S40, the omnivore may or may not lead to violation of the resulting set of assembly rules given by the $\tilde{n}_i$. In the resource-limited case, the requirement then becomes that interpretation of the omnivore as a consumer is possible, i.e. it must prey on one of the resource species levels (odd levels). Further, any of the inequalities in Eq. S38 must still hold when replacing with $\tilde{n}_i$. This would e.g. be impossible if the added species richness were to occur at a level already at the limit of the allowed number, e.g. when adding another top predator to a system where the number of top predators equals that of the bordering trophic level, $n_L = n_{L-1}$.

S6.1 Example of omnivory

Generally, multiple omnivores may be present in a given food web. We exemplify the effect of four omnivores ($n_g = 4$) on a possible configuration of diversities in a food web consisting of seven trophic levels (Fig. D). For simplicity, in the figure we consider all omnivores to prey on the same three trophic levels, 1, 4, and 6. Note also, that under absence of the omnivores, the food web presented in the figure violates the assembly rules, since $n_2 \geq n_1 - 1$ is not obeyed.

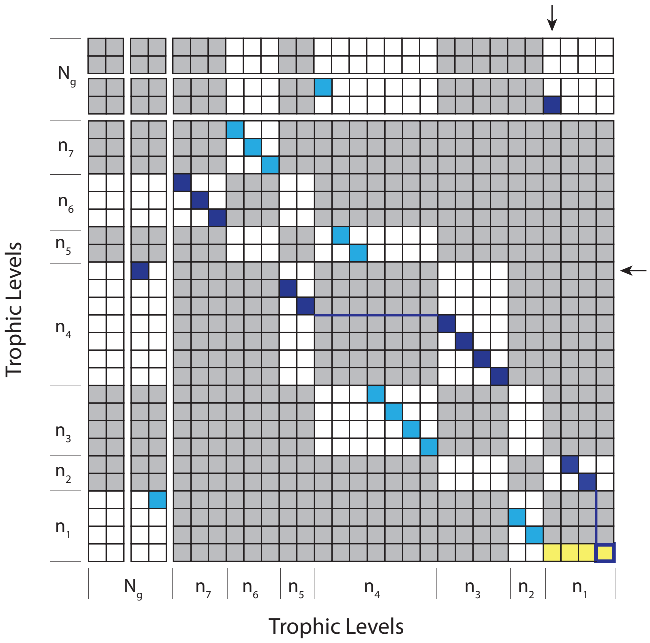

Figure D: **Inclusion of omnivory.** Interaction matrix for seven trophic levels with respective species diversities $n_1, \ldots n_7$. N_g specifies the number of omnivore species preying on more than one trophic level, here n_6, n_4, and n_1. The schematic shows that n_2 and n_5 do not fulfill the standard assembly rules but that the species richness at these levels can be supplemented by the omnivore count, i.e. $\tilde{n_2} \equiv n_2 + 1$, and $\tilde{n_5} \equiv n_5 + 1$, yielding $\tilde{N_g} \equiv N_g - 2$. The row and column of addition of these two omnivores is indicated by arrows. The two remaining omnivores can further be accommodated by interpreting one as further contributing to $\tilde{n_2}$, the other as contributing to $\tilde{n_5}$.

By interpreting one of the omnivores as a consumer of species at trophic level 1, the replacement $\tilde{n_2} \equiv n_2 + 1$ can be made. This modification allows the condition to be met, and we yield $\tilde{n_2} = n_1 - 1$. The subsequent condition $4 = n_3 \geq \tilde{n_2} - n_1 + 1 = 0$ is trivially fulfilled and $7 = n_4 \geq n_1 - 1 - \tilde{n_2} + n_3 = 4$ is also fulfilled in this example. However, at the next level, $2 = n_5 \geq n_4 - n_3 + n_2 - n_1 + 1 = 3$ is violated. Again, an omnivore can be interpreted to feed on a convenient trophic level, namely n_4, and thus allows the interpretation of this omnivore as a consumer at the level 5. This leads to the redefinition $\tilde{n_5} \equiv n_5 + 1$ and the assembly rule is now obeyed. The final two assembly rules involving n_6 and n_7 prove to be fulfilled.

At this stage of the analysis, two of the omnivores have been re-interpreted as trophic species allowing all assembly rules to be obeyed. However, the two remaining omnivores must also be accommodated without violation of the assembly rules. In the given example, this is possible by interpreting again one to reside at level 2, the other at level 5, all inequalities are still obeyed. Note that the possibility of accommodating multiple omnivores into an existing food web that is compatible with the assembly rules, is only possible when they allow interpretation as species on both even and odd trophic levels. This is the case for the example in Fig. D, where the omnivores prey on levels one and four.

S6.2 Generalist omnivory

The previous discussion shows that the presence of an omnivore allows more flexibility in interpreting the diversities n_i in the assembly rules (Eq. S38). The presence of an omnivore can hence allow for fulfillment of the assembly rules when a trophic species is added or removed, which would otherwise lead to a violation of the rules. The situation is most transparent for a generalist omnivore, which we define as a species that preys on all existing trophic levels. This type of omnivore allows greatest flexibility: (i) Incorporation into an existing food web is always compatible with the assembly rules as the generalist omnivore can either be interpreted as adding to N_o or N_e (which would then lead to an increase of N_o or N_e by up to the number of omnivores, yielding a modified $\tilde{N}_o$, respectively $\tilde{N}_e$). (ii) Upon addition of a new species into a food web encompassing the generalist omnivore, a possible violation of the assembly rules by the new species can often be circumvented by re-interpreting the generalist omnivore as a species residing on a convenient trophic level. Specifically, the assembly rules under presence of N_G generalist omnivores (subscript capital "G") in addition to the food web species richness described in the main text, are:

$$\sum_{l=1}^{k} \tilde{n}_l (-1)^{k+l+1} \begin{cases} \leq (-1)^k & \forall \quad k \in [1, L] \,, \text{ if } \tilde{N}_o - \tilde{N}_e = 1 \\ \leq 0 & \forall \quad k \in [1, L] \,, \text{ if } \tilde{N}_o - \tilde{N}_e = 0 \,, \end{cases} \tag{S41}$$

where $\tilde{N}_o - \tilde{N}_e \in \{0, 1\}$, $\tilde{n}_l \equiv n_l + \Omega_l$, $\Omega \equiv \sum_{l=1}^{L} \Omega_l \leq N_G$ and $\Omega_l \geq 0$ for all $l \in \{0, \dots, L\}$. This means that either of the $N_G \geq \Omega$ generalist omnivores can — but need not — be interpreted as an additional trophic species residing on any of the existing trophic levels.

S6.3 Pairing requirements for food-webs with omnivores

In terms of the pairing presented in Sec. S5, omnivores can allow additional pairing options as they prey on multiple trophic levels, making it easier to fulfill our basic assembly rule, $det(\mathcal{R}) \neq 0$ (compare Fig. 4, main text). It is however necessary to also consider an extension of the perfect matching criterion (Sec. S5), as omnivores in addition can open for nonzero determinants by the use of loops (or equivalently

cycles), see Fig. E. To discuss this we introduce the concept of *directed pairing*.

Each pairing of species that are connected, may influence the population sizes of each other (so far marked by a single solid arrow, Figs 1, 3 and 4, main text). To more explicitly highlight that the interaction influences both species involved in the pairing, we now use a more specific notation, where the single arrow is replaced by two dashed arrows (shown in Fig. E.a). Thereby, the interaction that allows species i to influence species j is marked with a directed dashed link from i to j. This may for example be a predator i that reduces the population of prey j, corresponding to a nonzero matrix element $\mathcal{R}_{ji}$. If the predator also gains in population by the interaction, there is a link from j to i, resulting in a nonzero matrix element $\mathcal{R}_{ij}$. A *directed pairing* is now defined as an interaction involving two species with only one direct link between them, i.e. either, but not both, of the two matrix elements $\mathcal{R}_{ji}$ and $\mathcal{R}_{ij}$. Pairings hence always involve two directed pairings in opposite directions (both matrix elements $\mathcal{R}_{ji}$ and $\mathcal{R}_{ij}$).

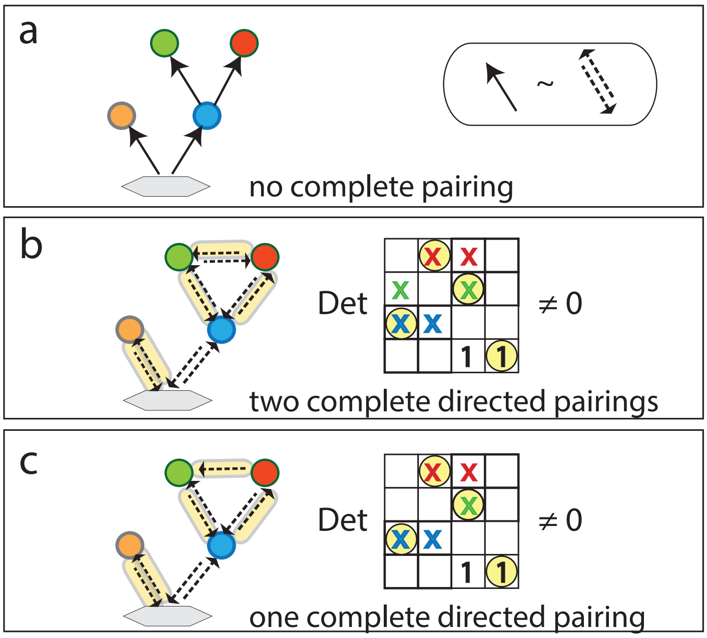

Figure E: **Pairing requirements for food webs involving omnivore species.** Example of a simple food web that can allow for coexistence, even if it is not completely paired. Yellow circles/ovals show interactions (links) that can be used to make a non-zero contribution to the determinant of $\mathcal{R}$.

Complete directed pairing: Consider a sequence of nonzero matrix elements $\mathcal{R}_{ij}$, $\mathcal{R}_{jk}$, $\mathcal{R}_{kl}, \ldots, \mathcal{R}_{mi}$. We call this type of sequence a *closed loop of directed pairings*, i.e. a chain of directed pairings where the direction is maintained and the last element connects to the first. When the entire food web is covered by non-overlapping closed loops of directed pairings, we refer to this as *complete directed pairing*. Each nutrient source can, but need not, participate as a species (and in this case each nutrient source participates in a bi-directional directed pairing with each basal species). $det(\mathcal{R})$ can only be nonzero

if the requirement of complete directed pairing is fulfilled. Note that a food web that can be perfectly matched will automatically allow a complete directed pairing.

Fig. E.a shows a food web that is not completely paired. The orange species needs to be paired with the resource, making it impossible to pair the remaining three species. Fig. E.b shows that the same food web can indeed be covered by complete directed pairings. Further, there are two permutations that can cover the food-web, the one shown and its mirror image for the closed loop of length three. For future use, Fig. E.c shows that even a network with uni-directional relations may be covered by a complete directed pairing. This is of relevance for later discussions of links from predators to the parasites of their prey (so-called concomitant links, see Sec. S9.3.3).

S7 Basal species with separate nutrients

So far we have restricted the discussion to the case of a single fundamental energy source that was shared by all primary producers. In practice, there may be several fundamental energy sources, such as different chemical compounds, in addition to sun light. Further, nutrient partitioning can also result from e.g. spatial subdivision or temporally varying availability (Tilman, 1994; McPeek, 1996; Tilman, 2000, 2011). As a consequence, a partitioning of the nutrient sources between the primary producers will occur. For simplicity, we take the n_1 primary producers to each depend on one of $n_S \leq n_1$ nutrients. In Eq. S1 this corresponds to setting only those p_{ij} equal to unity where i and j share a nutrient. In that case, $\mathcal{A}_0$ breaks down into a block structure with n_S blocks. Each of these blocks is a square matrix of unit entries and dimension equal to the number of primary producers sharing the corresponding nutrient. Each of these blocks can then be manipulated to yield a single row of ones and otherwise zeros. This means that n_S rows of unit entries persist. In the assembly rules the availability of the n_S nutrients again allows for more flexibility:

$$\sum_{l=1}^{k} n_l (-1)^{k+l+1} \leq \Delta(-1)^k \qquad \forall \quad k \in [1, L] \,, \tag{S42}$$

where $\Delta \equiv N_o - N_e \in [0, n_S]$. This set of conditions is analogous to the one for a single nutrient (Eq. S38) except that now, Δ can take any value between zero and the number of available nutrient types, a choice that then carries over to additional sets of inequalities.

S8 Species link requirement to include predator saturation

In consumer-resource relationships, type-II functional responses are characterized by a less-than linear increase of consumption as resource concentration grows. This distinguishes type-II from type-I responses where consumption is linear in resource concentration, i.e. $c(R) \sim R$, where c is the consumption rate per consumer, R is the concentration of the resource species and K is a constant. In the following we set the non-linear type-II response to

$$c(R) \sim R/(K+R) \tag{S43}$$

and discuss the resulting modifications of the food web assembly rules.

S8.1 Model equation with type-II response

Predator saturation, i.e. the situation where the predator growth rate saturates at a high prey density, can be taken into account in the assembly rules by replacing the type-I by the type-II functional response. For simplicity, let us again first consider the case of a completely shared nutrients for basal species and absence of omnivores. The population dynamics for the basal, respectively higher trophic levels, now obeys the following modified equations (compare Eqs 3 and 4, main text):

$$\begin{aligned} \frac{dS_i^{(1)}}{dt} &= k_i^{(1)} S_i^{(1)} \left[1 - \sum_j S_j^{(1)}\right] - \sum_k \tilde{\eta}_{ki}^{(2,1)} S_k^{(2)} \frac{S_i^{(1)}}{S_i^{(1)} + K_{ki}^{(2,1)}} - \alpha_i^{(1)} S_i^{(1)}, \text{ respectively} \\ \frac{dS_i^{(l)}}{dt} &= S_i^{(l)} \sum_j \beta_{ji}^{(l,l-1)} \tilde{\eta}_{ji}^{(l,l-1)} \frac{S_j^{(l-1)}}{S_j^{(l-1)} + K_{ji}^{(l,l-1)}} - \sum_k \tilde{\eta}_{ki}^{(l+1,l)} S_k^{(l+1)} \frac{S_i^{(l)}}{S_i^{(l)} + K_{ki}^{(l+1,l)}} - \alpha_i^{(l)} S_i^{(l)} . \end{aligned} \tag{S44}$$

Here, the term

$$\tilde{\eta}_{ki}^{(l+1,l)} S_k^{(l+1)} \frac{S_i^{(l)}}{S_i^{(l)} + K_{ki}^{(l+1,l)}} \tag{S45}$$

denotes the predation rate of the species k at the trophic level $l+1$ with respect to the species i at the trophic level l, with a half saturation density $K_{ki}^{(l+1,l)}$. When $S_i^{(l)} \ll K_{ki}^{(l+1,l)}$, the function will be reduced to the type-I response function as

$$\eta_{ki}^{(l+1,l)} S_k^{(l+1)} \cdot S_i^{(l)} \tag{S46}$$

with $\eta_{ki}^{(l+1,l)} \equiv \tilde{\eta}_{ki}^{(l+1,l)} / K_{ki}^{(l+1,l)}$, while when $S_k^{(l)} \gg K_{ki}^{(l+1,l)}$, the function will be reduced to the term

$$\tilde{\eta}_{ki}^{(l+1,l)} \cdot S_k^{(l+1)} , \tag{S47}$$

which implies that the species at level l is not fully exposed to the species population of $S_k^{(l+1)}$.

S8.2 Constraints from steady state condition and extended species pairing

The non-overlapping species pairing of nodes required for coexistence (described in the main text) can be extended to the type-II functional response case. In order to derive it, we first consider top predators, middle species, and basal species separately, and then summarize the extended rules.

S8.2.1 Top predators

Consider the top predators at the level L. Their population dynamics is given by

$$\frac{1}{S_i^{(L)}}\frac{dS_i^{(L)}}{dt} \quad = \quad \sum_j \beta_{ji}^{(l,l-1)}\tilde{\eta}_{ji}^{(L,L-1)}\frac{S_j^{(L-1)}}{S_j^{(L-1)}+K_{ji}^{(L,L-1)}} - \alpha_i^{(l)}. \tag{S48}$$

Assuming $S_i^{(L)} \neq 0$, the steady state condition gives

$$\sum_j \beta_{ji}^{(l,l-1)}\tilde{\eta}_{ji}^{(L,L-1)}\frac{S_j^{(L-1)}}{S_j^{(L-1)}+K_{ji}^{(L,L-1)}} - \alpha_i^{(l)} = 0\ . \tag{S49}$$

Eq. S49 amounts to one constraint among the populations' of the all of its prey species $S_j^{(L-1)}$. For n_L top predator species in steady state, it must be ensured that Eq. S49 does not over-constrain the densities of prey populations. There should hence be n_L prey species whose populations are determined by these equations. In other words, it should be possible to pair each top predator with one of its direct prey species in a non-overlapping way. Hence, for top predators and their prey, the pairing requirement remains unchanged, irrespective of whether the type-I or type-II response is of interest.

S8.2.2 Intermediate species

Now consider a species i at a trophic level l, that is neither a top predator nor a basal species.

Type-I response of all predators with respect to $S_i^{(l)}$ — Suppose the population $S_i^{(l)}$ is small enough to satisfy $S_i^{(l)} \ll K_{ki}^{(l+1,l)}$ for all of its predator k; for convenience, we term such species "type-I response species". The dynamical equation for the population density of $S_i^{(l)}$ will become

$$\frac{1}{S_i^{(l)}}\frac{dS_i^{(l)}}{dt} = \sum_j \beta_{ji}^{(l,l-1)}\tilde{\eta}_{ji}^{(l,l-1)}\frac{S_j^{(l-1)}}{S_j^{(l-1)}+K_{ji}^{(l,l-1)}} - \sum_k \tilde{\eta}_{ki}^{(l+1,l)}S_k^{(l+1)} - \alpha_i^{(l)}. \tag{S50}$$

In this case, with assuming $S_i^{(l)} \neq 0$ the steady state of Eq. S50 gives one constraint among its predators' population $S_k^{(l+1)}$ and its preys' population $S_j^{(l-1)}$. In order that this condition does not over-constrain the populations, it should be possible to pair all the type-I response species either to one of its predators or one of its prey species in a non-overlapping way. This pairing requirement for the top predators and their prey species hence remains unchanged as compared to purely type-I response consumer-resource

relationships (compare main text).

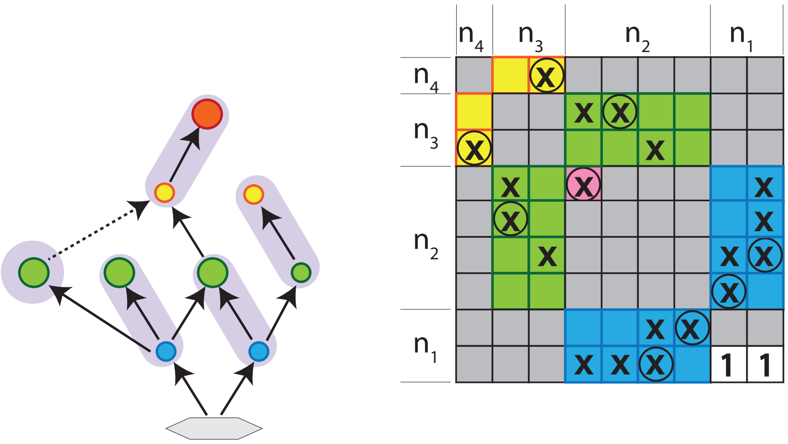

Figure F: **Simple food web containing a type-II response species.** The figure is an extension of Fig. 3a, main text, where an additional species was included at trophic level 2, i.e. now $n_2 = 4$. This species is taken to be in the saturation phase w.r.t. the response to predation by a predator on trophic level 3 (the type-II response is indicated by a dotted arrow). Under these circumstances, the species can be considered to pair with itself in the species pairing, indicated by a shaded circle. When viewing the adjacency matrix as a table of relations between a consumer and a resource species, the self-pairing could be interpreted as diagonal matrix element (shown as a pink square), i.e. a dependency of the change in species density on itself.

Type II response of some predators with respect to $S_i^{(l)}$ — Suppose $S_i^{(l)}$ is comparable or larger than at least one of the constants $K_{ki}^{(l+1,l)}$; we term such interactions "type-II response". The equation will then take the form

$$\begin{aligned}\frac{1}{S_i^{(l)}}\frac{dS_i^{(l)}}{dt} &= \sum_j \beta_{ji}^{(l,l-1)}\tilde{\eta}_{ji}^{(l,l-1)}\frac{S_j^{(l-1)}}{S_j^{(l-1)}+K_{ji}^{(l,l-1)}} \\ &\quad -\sum_{k'}\eta_{k'i}^{(l+1,l)}S_{k'}^{(l+1)}-\sum_k \tilde{\eta}_{ki}^{(l+1,l)}S_k^{(l+1)}\frac{1}{S_i^{(l)}+K_{ki}^{(l+1,l)}}-\alpha_i^{(l)}. \qquad \text{(S51)}\end{aligned}$$

In this case, given $S_i^{(l)} \neq 0$, the steady state of Eq. S50 yields one constraint among its own population $S_i^{(l)}$, its predators' populations $S_k^{(l+1)}$, and its prey species' populations $S_j^{(l-1)}$. For this condition not to over-constrain the populations, it should now be possible to link each of the type-II response species to either itself, one of its predators, or one of its prey species in a non-overlapping way.

An example of a food web involving a type-II response species is shown in Fig. F, demonstrating the possible self-pairing for the added species given that the saturation regime is reached. Note that the ability of a species' self-pairing comes with the condition that the saturation level K in Eq. S43 is reached by the species population density. In the example given in Fig. F this may impose relatively strict requirements on K since its resource species (shown in blue) is already in a consumer-limited state (small circle radius). Recent work has explored different functional responses as well as different types of interaction (competitive vs. mutualistic). It was found that combining a range of responses can have a

stabilizing effect on communities (Mougi and Kondoh, 2012; Brose et al., 2006).

S8.2.3 Basal species

Type-I response of all predators with respect to $S_i^{(1)}$ — If $S_i^{(1)} \ll K_{ki}^{(2,1)}$ for all its predator k, a basal species obeys the equation

$$\frac{1}{S_i^{(1)}}\frac{dS_i^{(1)}}{dt} = k_i^{(1)}\left[1 - \sum_j S_j^{(1)}\right] - \sum_k \eta_{ki}^{(2,1)} S_k^{(2)} - \alpha_i^{(1)}. \tag{S52}$$

The steady state condition with $S_i^{(1)} \neq 0$ gives a constraint among the sum of basal species $\sum_j S_j^{(1)}$ and its predator population $S_k^{(2)}$'s. Hence, for this condition not to over-constrain the populations, it should be possible to pair each of the type-I response basal species to one of its predators in a non-overlapping way, allowing additionally one of the basal species to pair with the nutrient source.

Type-II response of some predators with respect to $S_i^{(1)}$ — The equation now becomes

$$\frac{1}{S_i^{(1)}}\frac{dS_i^{(1)}}{dt} = k_i^{(1)}\left[1 - \sum_j S_j^{(1)}\right] - \sum_{k'} \eta_{k'i}^{(2,1)} S_k^{(2)} - \sum_k \tilde{\eta}_{ki}^{(2,1)} S_k^{(2)} \frac{1}{S_i^{(1)} + K_{ki}^{(2,1)}} - \alpha_i^{(1)}. \tag{S53}$$

The steady state condition with $S_i^{(1)} \neq 0$ gives a constraint among its own population $S_i^{(1)}$, the sum of basal species $\sum_j S_j^{(1)}$ and its predators' populations $S_k^{(2)}$.

In order for this condition not to over-constrain the populations, it should be possible to pair each of the type-II response basal species to either itself or one of its predators in a non-overlapping way, allowing again one of the basal species to pair with the nutrient source. The generalization to multiple nutrient sources is analogous to the discussion in Sec. S7, i.e. each nutrient source may, but need not, be used for pairing a a basal species.

S8.2.4 Summary of the extended species pairing requirement

Summarizing the conditions above, the species pairing requirements with type-II response read as follows:

- It must be possible to produce a pairing of each species and one of its "partners" in a non-overlapping way.
- This partner can be any of its predator or prey species if the species' response to all its predators is in the type-I regime (sufficiently low species density).
- If a species' response to one or more of its predators are not in the regime of type-I response (sufficiently high species density), it can be paired to itself. This self-pairing corresponds to a self-limit on growth which prevents it from fully controlling its prey.

- If the species is a basal species, each nutrient source is also a possible partner.

S9 Data Analysis

This section describes details of the data analysis performed. In particular, we describe the characteristics of the food web data considered (Sec. S9.1), an analysis of the sub webs formed by free-living species only (Sec. S9.2) as well as the analysis of the sub webs including parasite species (Sec. S9.3). The latter also involves the discussion of concomitant predation (Sec. S9.3.3). Several of these sections involve specific comments on the individual food webs, we have noted this by "details", to facilitate reading. We have also moved more detailed figures (included for completeness) to the end of the text. We further examine the possible existence of secondary extinctions by removal of species in the different empirical food webs (Sec. S9.4).

S9.1 Data considerations

S9.1.1 Food web data

We use high-resolution data on seven food webs including free-living and parasite species: The North American Pacific Coast webs Carpinteria Salt Marsh, Estero de Punta Banda, Bahia Falsa (Hechinger et al., 2011; Lafferty et al., 2006); the coastal webs Flensburg Fjord (Zander et al., 2011), Sylt Tidal Basin (Thieltges et al., 2011), and Otago Harbor, New Zealand (Mouritsen et al., 2011), as well as the Ythan Estuary, Scotland (Huxham et al., 1996). These food webs describe consumer-resource interactions between basal, predatory and parasite species. A compilation of all seven food webs has recently been provided (Dunne et al., 2013). Specifically, the data distinguish three types of links: (i) links between free-living species only ("Free"), (ii) additional links between parasites and other species ("Par") and (iii) links from free-living consumers to the parasites of their resources ("ParCon"), i.e. so-called concomitant links.

S9.1.2 Technical considerations on parasites

Similar to predators, parasites feed on their hosts. Parasite-host interaction can be distinguished from predator-prey interactions in that parasites undergo complex life cycles (Huxham et al., 1996), where a single parasite at one stage may benefit from one host but a different host at another stage. In addition, a parasite often lives in or on its hosts, and concomitant links from the hosts' predators' to the parasite may result (Sec. S9.3.3 and Sec. S10.4). On the population level, the total population of a given parasite species can however be regarded as feeding on a number of different hosts simultaneously. We adopt this view in the following as far as "Par" is concerned, as the details do not impact on our mathematical description in terms of food web matrices. However, these simplifications should be kept in mind when considering, in particular, removal of species and the question of potential sustainability for the resulting webs (Sec. S9.4). When considering concomitant links, we make additional distinctions (Sec. S9.3.3). For

example, one aspect, which we further consider, is that of the host potentially also being impacted upon by the parasite, e.g. by reduction or modification of activity, thereby affecting metabolism or reproductive success (Lafferty and Morris, 1996; Johnson et al., 2010).

S9.1.3 Matrix rank, basal species and nutrient sources

For our general coexistence condition of $\det(\mathcal{R}) \neq 0$ we do not require details of the numerical values of matrix coefficients. Instead, we first symmetrize the interaction matrix to mimic the bi-directional effect in predator-prey, respectively parasite-host pairs. Further, we allow basal species to feed on individual ($p_{ij} = \delta_{ij}$). We then assign random numbers to all nonzero matrix elements to numerically compute the determinant. To allow a more refined view on food webs exhibiting $\det(\mathcal{R}) = 0$ we have computed the rank r of $\mathcal{R}$ (Otto and Day, 2007). r describes the number of linearly independent rows (or columns) of the matrix. Ecologically, the rank amounts to the number of uniquely occupied niches in the ecology. We also repeatedly use the *rank deficiency* (i.e. nullity) $d \equiv S - r$, with S the total number of species. We distinguish d_f, d_{f+p}, $d_{con,asym}$, and $d_{con,sym}$, the deficiencies of the sub webs of free-living, free-living and parasite species, as well as additional asymmetric and symmetric concomitant links (Sec. S9.3.3).

S9.1.4 Treatment of detritus and particulate organic matter (POM) in the data

Several of the empirical food web data sets contain detritus or POM as basal species (Wilson and Wolkovich, 2011; Gaedke et al., 1996; Pauly, 1996). The equation describing the concentration of such non-alive "basal species" D_i should have a source term that is independent of D_i, namely

$$\frac{1}{D_i}\frac{dD_i}{dt} = \frac{k_i^{(D)}}{D_i} - \alpha_i^{(D)} - \sum_{k=1}^{n_2} \eta_{ki}^{(2,1)} S_k^{(2)}. \tag{S54}$$

The first term on the RHS is not linear in D_i, therefore the equation for the steady state is not expressed by the matrix form. However, as summarized in section S8, the species pairing can be extended to the nonlinear equation as a condition not to over-constrain the variables. In this regard, the steady state of Eq. S54 constrains its own "population" D_i and its consumers' populations $S_k^{(2)}$. Thus, the D_i can be paired with either themselves or one of their consumers, as in the case of a basal species with its own nutrient. We therefore treat such resources as basal species with their own nutrient in the data analysis.

S9.1.5 Approximate trophic levels

For all empirical food webs, we first organize species according to their approximate trophic level. In accordance with the literature (Williams and Martinez, 2004; Thompson et al., 2007), we here aim to define the trophic level of a given species as its average food chain length. Basal species, which do not consume other species, are thereby assigned trophic level 1. Within the prey-averaged definition (Williams and Martinez, 2004), the trophic level of a given species is then the average trophic level of all its resource

species plus one, e.g. a species that only consumes basal species has trophic level 2. In practice, we have determined chain lengths by following paths from a given species to one of the basal species, choosing a random resource at equal probability at each intersection. As choices are random, we have repeated the procedure to ensure that fluctuations are small and the general conclusions do not depend on the sampling. For two species with very similar approximate trophic levels the order could occasionally switch, an effect that we were able to reduce by using larger sample size. We compared results using between 2×10^3 and 2×10^4 food chain samples per species and detected very little variation. Some food webs contain loops, where the same species recurs within a single food chain. These loops were dropped from the analysis on trophic levels. In the following, we use the computed average and standard deviation of a species' food chain lengths as a proxy its trophic position and degree of omnivory, respectively. A similar consideration has been made in the literature (Lafferty and Morris, 1996). We will, for simplified syntax, in the following refer repeatedly to "trophic level" when discussing species, by which we mean the approximate average food chain length of the given species.

S9.2 Analysis of food web matrices of free-living species

S9.2.1 Trophic levels and omnivory

For all food webs, we compute the frequency distributions of average food chain lengths. We also aggregate the data for all food webs and produce the corresponding frequency distribution (Fig. G). Notably, the distribution of aggregated empirical data (Fig. G.a) has pronounced, sharp peaks at chain length 1 and 2, corresponding to basal species and their consumers. Approximately 25 percent of species are sharply located at trophic level 2 (dashed red line in the plot). The absolute highest average chain length lies near 4.5 (compare also to the literature, e.g. (Thompson et al., 2007)). Comparing with the individual food webs (Fig. V), this overall pattern is generally preserved. In both figures (Figs G and V), we compare to data produced using the niche and cascade models, with total species number S and total number of links L for each food web as an input. These models generally produce fewer species at the level 2 and overall considerably broader distribution functions ranging up to chain lengths of 5.5.

To quantify the extent to which species can be associated with sharp trophic levels, we compute also the standard deviation of food chain length for each species, and show the aggregated distribution functions for all resulting values (Fig. N.a—c). We hereby define a species to have a "sharp trophic level", if the species shows no variation, i.e. zero standard deviation, in food chain length. In the plots (Fig. N.a—c), a value of zero correspondingly means that all chain lengths associated with a given species are identical. The figure demonstrates that in the empirical data (Fig. N.a), all food webs have a considerable fraction (approximately 40 percent on average) of species with sharp trophic levels. The remaining species have chain length standard deviations of generally less than one. A value of $\sim 1/2$ would e.g. be obtained by

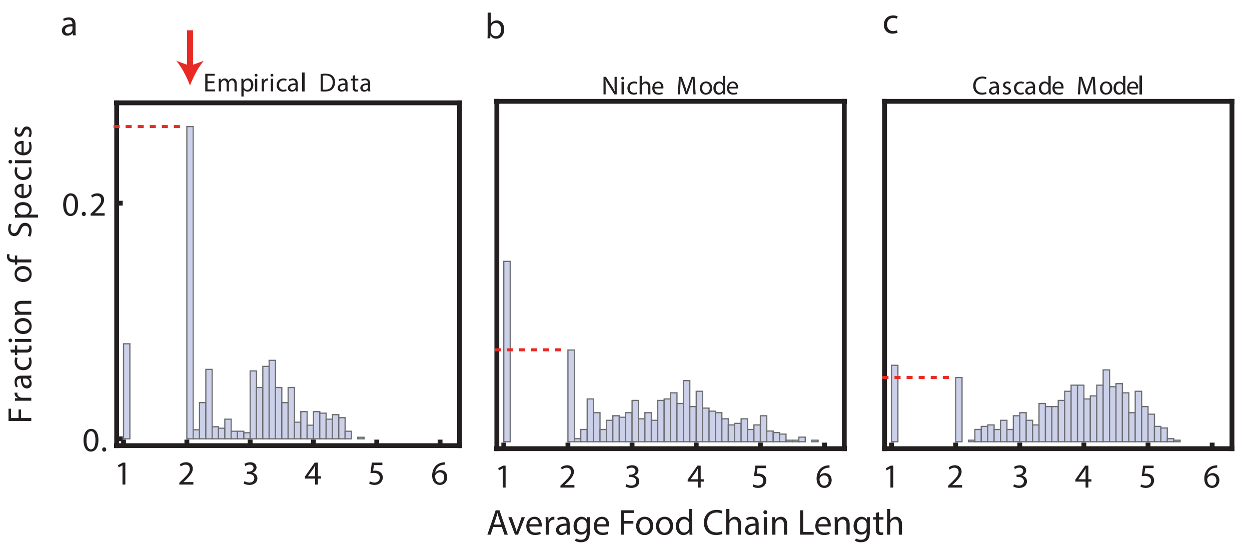

Figure G: **Average food chain length distribution (aggregated for all webs). a**, Histograms of species' average food chain lengths when aggregating species of all seven food webs. **b, c**, comparison with niche and cascade model, respectively. Red arrow highlights the spike formed by level 2 species in the empirical data. dashed horizontal lines are guides to the eye, illustrating the respective concentrations of level 2 species in the three cases.

a species that consumes at two neighboring trophic levels equally. In comparison, the niche and cascade models (Fig. N.b,c) predict substantially fewer sharp trophic levels. Further, they produce a continuum of values ranging up to 2, which could e.g. be obtained by a species consuming equally on trophic levels 1 and 5. These aggregated results should also be compared to the distributions for the individuals webs (Fig. W), where the general pattern is preserved, albeit with some variation (which we will return to in Sec. S10).

S9.2.2 Trophic structure of food web matrices

We now use the obtained mean chain length (CL) of each species to assign an order to species from shortest to longest CL. Using the Bahia Falsa food web as an example, we describe the resulting depiction of food webs (Fig. H, caption). We will subsequently employ this presentation style repeatedly in the remaining text. We also use integer-rounded CL to broadly categorize species (the CL is thereby simply rounded to the nearest integer value). To simplify the discussion, in the following, we refer to interactions between species with integer-rounded chain length l_1 and l_2 as $l_1 - l_2$ interactions. Consider now the corresponding depiction of all empirical food web matrices of free-living species (Fig. T).

Additional detail. We here focus first on the sub web of free-living species only. All food webs show a clear structure of blocks of high link density and blocks where links are generally absent. We have in Sec. S9.2.1 noted that species of integer-rounded CL 2 have little variation in chain length. This is also evident from the figure, as the positions of the white blocks in the matrices indicate that species of CL 2 predominantly consume basal species and are in turn mainly consumed by species of CL 3. We further find that the structure of the matrices becomes less defined for species of CL 3 and above. There, different food webs show different behavior. Generically, more links can be observed between species of similar chain lengths, e.g. in the block with chain lengths ~ 3 considerable interaction is present, while

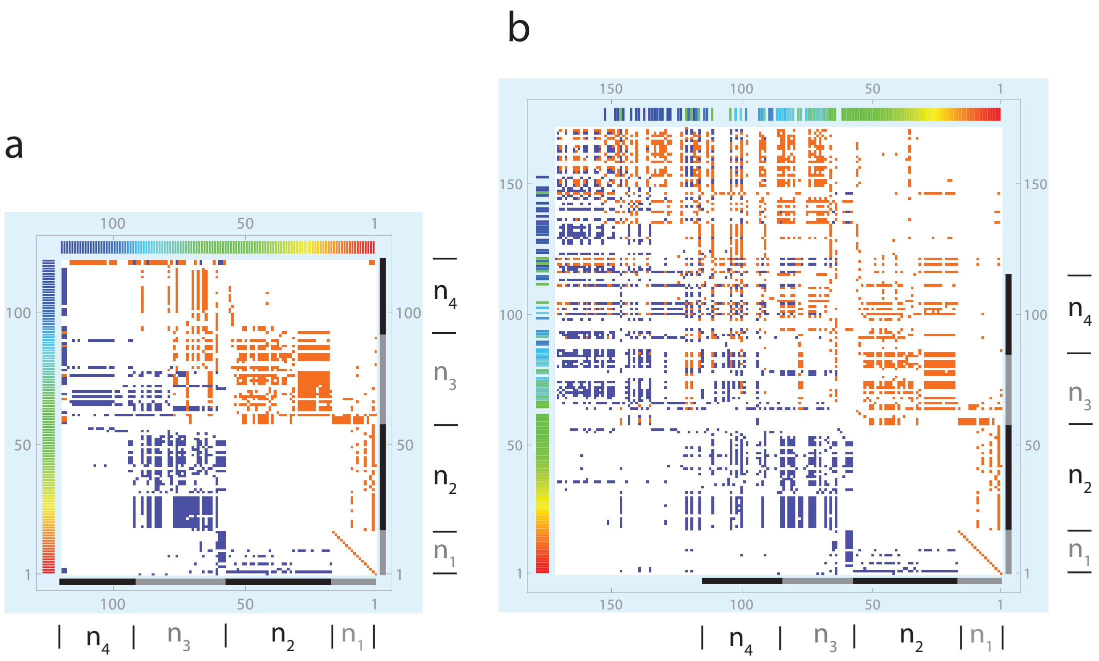

Figure H: **Depiction of Bahia Falsa food web matrix using ordering by approximate trophic levels.** **a**, Free-living web consisting of 119 species. **b**, Web of free-living and 52 parasite species. Orange and blue colors of matrix elements represent the direction of interaction between species. Color-coding shown at top and left edge of matrix indicates the ordering of species in terms of their average food chain length (red=short, blue=long). This color coding is used in (b) to indicate the new positions of free-living species once parasites are introduced (there is little re-ordering for the lowest 60 species, but more re-ordering above). Gray and black "measuring bars" at the lower and right edge of the food web matrices indicates the number of integer-rounded approximate trophic levels and corresponding diversities n_i. These measuring bars can be used to compare the structure of the matrices in (a) and (b). Basal species have again been assigned individual nutrient sources, yielding finite matrix elements on the diagonal in the lower right block matrix. Figure is a reproduction of main text Fig. 5a,d.

this is e.g. absent for species with CL 2 (white spaces). These matrices show additional detail on the trophic organization of species: In addition, a clear hierarchical structure is evident, illustrated by blue and red colors (corresponding to the direction of the consumer-resource links). Species located at higher trophic levels tend to act as consumers of species below them.

S9.2.3 Discussion of individual matrices (details)

Using Fig. 1, main text, we attempt to construct a path through the matrices. For the different matrices, we make the following observations:

- Bahia Falsa: Constructing a path is not possible as a large number of well-defined chain length 2 species has formed a white block that is impossible to circumvent using the available 2-3 interactions (interactions between species of integer-rounded CL 2 and 3). This could be remedied by the presence of 4-2 interactions, but also these are mainly absent. In this particular food web, the interactions 4-4 are also generally missing (there is only a thin band of omnivores feeding on levels 3 and 4). Hence, the Bahia Falsa food web constitutes a relatively well-structured food web and the high degree of organization, together with a large number of CL 2 species leads to failure to comply with the assembly rules (the rank difference — i.e. nullity — is $d_f = 16$, compare the table in Fig. Md).

- There are two other food webs with clear structure: Flensburg Fjord and Otago Harbor. Both have nearly complete lack of 2-2 interaction and little 2-4 interaction. Both have large 2-2 blocks. Also in these food webs, the assembly rules can not be fulfilled, the respective rank differences are 15 and 19.

- A further food web with large rank difference is Ythan Estuary ($d_f = 17$). Here, however, the overall link density is very low, compared to the other food webs, leading to an additional difficulty in reaching agreement with the assembly rules.

- The remaining food webs Carpinteria, Punta Banda, and the Sylt Tidal Basin are less clearly structured and the 2-2 block is somewhat smaller. Correspondingly, a path through the matrix can be constructed for Carpinteria and Punta Banda ($d_f = 0$). Only a small rank difference results for Sylt Tidal Basin ($d_f = 6$).

Again, we compare with the simulations obtained from the niche and cascade models.

- Both models describe the hierarchical organization of species (clear organization of blue and red matrix elements), where those with larger CL prey on those with shorter CL. This is clear by the definition of the two models, where an ordering of species is the starting point.

- Both models deviate from the empirical data in that they do not produce any obvious signature of trophic levels, i.e. no block structure is apparent in either of the two models.

- Correspondingly, for all instances of the models considered (with the empirical numbers of species and links), full rank was achieved. Indeed, for random matrices containing exclusively values -1, 0, and 1, with relatively mild conditions, one is very likely to achieve full rank (Tao and Vu, 2006).

- We have further reduced the number of links considerably for the two models: Demanding only that each species be connected to at least one other species, essentially all (> 99 percent) of samples achieved full rank, even when using only 2/3 of the links. In the remaining few cases, a rank difference of 1 resulted.

S9.3 Analysis of food web matrices including parasite species

S9.3.1 Trophic positions with parasites

We now consider the empirical data also for food webs including parasites (Fig. U). We aim to characterize the apparent re-organization that takes place once parasites are included in the food web. We again make use of approximate trophic positions, but it should be kept in mind that for parasites trophic positions are a matter of debate, as they undergo complex life-stages and localizing them at any specific trophic level (or assigning a specific food chain length) is questionable. Here, we use the concept of trophic

levels, respectively food chain length, solely as a means to organize parasite species relative to free-living species and discuss the respective host range. To this end, we use a color-coding (displayed at the edge of the food web matrices in the figure and explained in Fig. H). For the free-living food webs, the color coding simply indicates the sequential organization in terms of the chain length computed for the free-living species alone. When parasites are included, the sequence of the color coding becomes disrupted. However, the disruption is again far from random: Nearly all species with CL 1 and 2 have unchanged positions in the extended food webs. For species with CL 3, some disruption takes place, i.e. some parasites now have chain lengths ~ 3. However, most of the parasites enter near the top end of the existing chain lengths and top-predators of the free-living web often no longer have largest chain length in the combined system (Lafferty et al., 2006). This feature of disruption is observed generally for all food webs studied. We also show distribution functions of the respective chain lengths in Fig. AA.

We note more specific features of the matrix structure: Parasites have re-organized parts of the structure at higher trophic positions, leading to substantial randomization there. However, much of the structure in the lower part of the matrix is left intact, in particular, the white block formed by 2-2 interactions is nearly unchanged in most of the food webs. Another noteworthy observation is that the hierarchical structure, highlighted earlier for the free-living webs, is strongly disrupted for the higher trophic levels, when parasites are considered (blue and red matrix elements are now more mixed).

S9.3.2 Discussion of individual food webs (details)

Here it is important to consider the different food webs separately (Fig. U): Carpinteria and Punta Banda show a strong mix-up of hierarchy for the higher levels, while this effect is less clear for some of the other food webs. In particular, Ythan Estuary shows nearly no reduction of the hierarchical structure. Mixing of hierarchical structure is important as it can lead to an increase in chain length (e.g. free-living species of formerly short chain length can sometimes feed on parasites that then feed on others, thereby increasing chain length). This particular feature is most evident when again considering the distributions of chain length standard deviation (Fig. Z) or the coefficient of variation (Fig. S). Carpinteria and Punta Banda have now acquired much stronger variation of food chain length for some species, values that now even exceed those produced by the corresponding Niche and Cascade model simulations. In the other extreme, the Ythan Estuary food web, the unbroken hierarchical organization leads to nearly unchanged chain length variation distribution. For completeness, we present also the aggregated data of chain length standard deviation for the case of free-living and parasite species (Fig. Y).

The above findings on chain length standard deviation and mixing of hierarchical structure are also consistent with the changes in chain lengths (Fig. X, compare Lafferty et al. (2006)). Here, it is found that a continuum of chain lengths beyond 3 ranging up to 5 or 6, in some cases, is now present. However, the pronounced concentration of level 2 species is conserved (most evident in the aggregated data, Fig. G

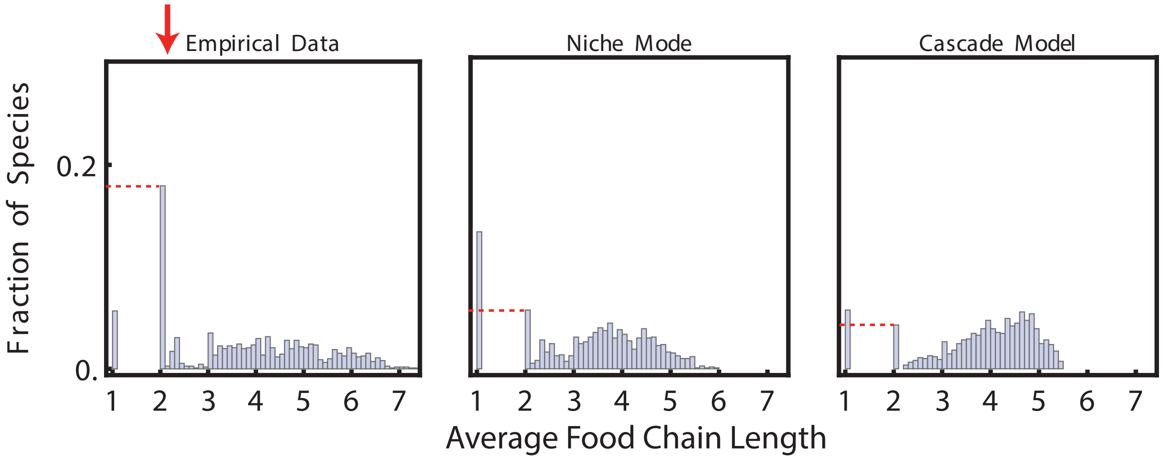

Figure I: **Aggregated chain length distributions for food webs including parasite species.** Similar to Fig. G but for all data including parasite species.

and Fig. I). This means, that the disruption, caused by the presence of parasites, affects mainly the upper part of the food web and introduces re-organization there. The lower part of the food web remains nearly unchanged.

Returning to Fig. U, we can discuss the relation to the assembly rules. For the Bahia Falsa web, some parasites prey on level 2, granting better chances to find a path through the food web matrix as demanded by the assembly rules (Fig. 1, main text). Indeed, in the Bahia Falsa food web, presence of parasites reduced the nullity from $d_f = 16$ to $d_{f+p} = 3$. Similar behavior is observed for Otago Harbor, where substantially more species now prey on level 2 species. On the other hand, in the case of the Ythan Estuary, not much structure is added and overall low link density makes finding a path less likely. This may be one reason why the nullity in fact increases when including parasites (others are discussed in Sec. S10).

S9.3.3 Concomitant predation

We further consider concomitant links: Concomitant links are links from a free-living consumer to the parasites of its resources (Lafferty et al., 2008, 2006), e.g. by consuming the resource, also the parasite will be consumed, causing an additional death rate to the parasite. Such links have e.g. been suggested to contribute considerably to parasite mortality (Johnson et al., 2010). Specifically, in the following we distinguish asymmetric and symmetric concomitant links.

Asymmetric concomitant effects arise, when a predator causes death to its prey's parasite(s), but no effect of the consumption of the parasite is felt by the predator. Generally, this is a type of 3-species interaction, which can be a function of all three species involved (which is beyond the simple products of the generalized Lotka-Volterra equations). In principle, one could e.g. include terms of the type:

$$\frac{d[parasite]}{dt} \sim \ldots . - \eta^* \cdot [predator] \cdot [host] \cdot [parasite] + \ldots\ , \tag{S55}$$

where η^* is a modified interaction probability. We here make the point that such interactions can lead to additional niches, as they impose new relations between parasites and the hosts' predators. To consider the presence of such interaction, it is here therefore sufficient to consider only the case where the coupling between the parasite and the host's predator does not vary with host density:

$$\frac{d[parasite]}{dt} \sim \ldots . - \eta^{**} \cdot [predator] \cdot [parasite] + \ldots \ , \tag{S56}$$

implying a linear truncation. (Non-linear interactions could also be considered in analogy to Sec. S8.) In terms of the interaction matrix, this is a directed link where the parasite feels the negative effect of the host's predator.

In such cases, the interaction matrix has a nonzero entry at some position (i, j) (Fig. Ec). The entry at the symmetric position (j, i) might be zero, if the predator's gain resulting from consumption of its prey does not change when the prey is infected by the parasite. For food webs with some uni-directional links, the basic condition $det(\mathcal{R}) \neq 0$, i.e. "finding a path through the matrix", amounts to the requirement of possible selection of an out-link and an in-link for each species in the interaction matrix.

Fig. Ec illustrates how directed links can provide sustainability: In the case depicted, the parasite (green) is exposed to concomitant predation by the predator (red). Without the concomitant link, this food-web would violate the competitive exclusion principle. In fact the rank deficiency of the interaction matrix would be $d = 1$ because the parasite and the predator compete. However, when one includes one directed concomitant link between predator and parasite, $d = 0$. This reduction in nullity comes about through inclusion of the cyclic interactions

$$predator \ \rightarrow \ parasite \ \rightarrow \ prey \ \rightarrow \ predator \tag{S57}$$

corresponding to the entries marked with yellow circles in the matrix on the right side of Fig. Ec.

Fig. E emphasizes that directed concomitant links can decrease the nullity of the interaction matrix. However, they will not always do so. In practice, when assuming that all concomitant links are directed, the nullity is only improved in two out of the seven food webs (Ythan Estuary and Otago Harbor), see the labels in the panels of Fig. J. In the remaining five food webs the nullity remains unchanged. Although directed links may help, one should realize that the use of loops constrains the pairing of other species. Thus, when the pairing involves one directed interaction, complete directed pairing requires a closed cyclic loop of reactions, involving species that then cannot participate in another pairing.

Fig. J examines the number of loops of length three ("triangles") for the seven food webs, with and without parasites. The panels also show the nullity of the corresponding food web. The figure illustrates that absence of rank gain from directed concomitant links is associated to food webs with many loops.

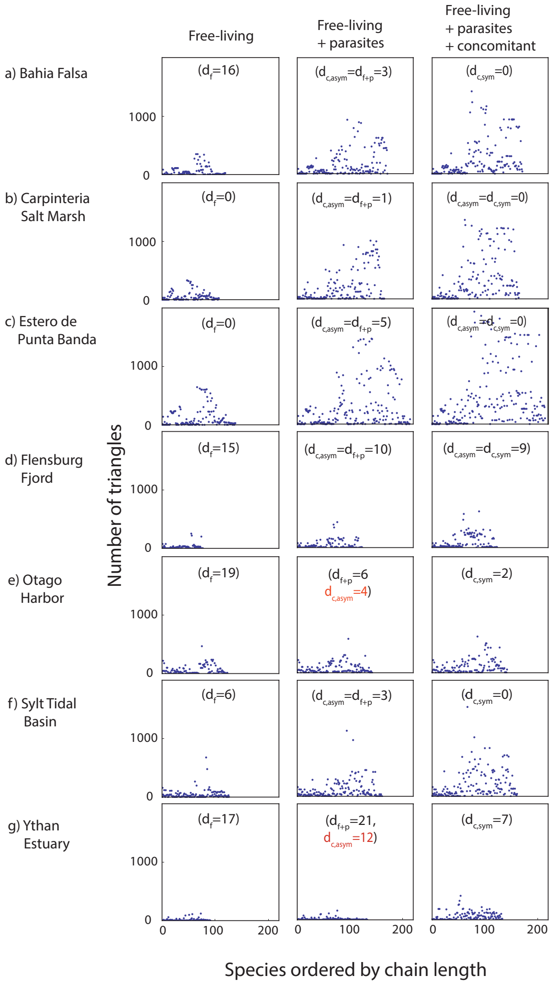

Data set	all free-living no para	all free-living para	S_f	S_p
Bahia Falsa	41	78	119	52
Carpinteria	23	84	107	58
Punta Banda	40	98	138	76
Flensburg Fjord	33	44	77	46
Otago Harbor	45	78	123	19
Sylt Tidal Basin	42	84	126	35
Ythan Estuary	45	46	91	42

Data set	odd levels no para	odd levels para	even levels no para	even levels para
Bahia Falsa	9	42	32	36
Carpinteria	8	39	15	45
Punta Banda	11	48	29	50
Flensburg Fjord	12	17	21	27
Otago Harbor	18	26	27	52
Sylt Tidal Basin	7	40	35	44
Ythan Estuary	26	15	19	31

Figure J: **Characterization of concomitant links.** Numbers of loops of length three (triangles) of each species for all food webs (as labeled), for the sub-webs of free-living, free-living and parasite links, as well as additional symmetric concomitant links. Loops were computed by first removing diagonal elements from $\mathcal{R}$ and collecting the diagonal elements of the cube of the resulting matrix. Note that we count each existing closed loop only once, irrespective of whether the loop may be directed or bi-directional. Panels mark the rank deficiencies for all cases, red labels highlight where changes occur for asymmetric concomitant links. Plot also shows the frequency distribution for numbers of free-living species with parasites, distinguishing additionally the set of high level (i.e. at least CL 3) free-living species. The table shows the numbers of free-living species that interact with parasites (i.e. either act as parasite hosts or feed on parasites, "para"), and do not interact ("no para"). Also shown: numbers of free-living and parasite species, as well as the numbers for interaction with free-living species at odd and even levels, respectively.

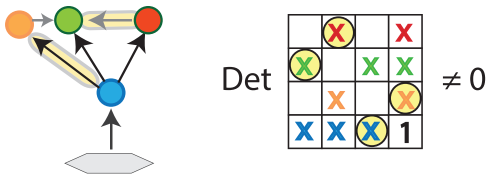

Figure K: **Possible pairing for symmetric concomitant links.** Food web consisting of host (blue), two predators of the host (orange, red) as well as a parasite (green). Consumer-resource (concomitant) links are shows as black (gray) arrows. Matrix shows the existing interactions and a possible path through the matrix (circles). The food web is sustainable with symmetric concomitant links, but not without.

In contrast, food webs with few loops (Ythan Estuary and Otago Harbor) open for a decrease in nullity by inclusion of these directed concomitant links.

Symmetric concomitant effects. The presence of a parasite may in some cases have effective impact on the consumption rate (Johnson et al., 2010). For example, by altering host behavior, the parasite may facilitate consumption of its host by the consumer, as was shown for killifish that become more susceptible to their natural enemies (birds) when they were infected by trematodes (Lafferty and Morris, 1996).

In such cases, the fraction of hosts infected by a parasite would have a larger probability to be consumed by the predator. This again constitutes a non-linear 3-species interaction as in Eq. S55. Again making a linear approximation, where the predator gain now linearly increases with parasite density, we then consider symmetric concomitant links. These links involve a bi-directional interaction between the host's parasite and predator (see Fig. K for a simple example where coexistence can be obtained). However, in all empirical cases, the full food webs with parasites and bi-directional concomitant links have lower nullity than the food webs without parasites. We have explored in the empirical data, whether only a small subset of bi-directional concomitant links is sufficient, finding that usually 20 percent yield already substantial improvement of the rank (compare Fig. 5f, main text). Four out of seven full food webs fulfill our assembly rule. Concomitant links are further explored in the simulations in Sec. S10.

S9.4 Removal of species from food web

The lack of niches d can be used to address the potential effect of removal of one species from a food web. If removal increases the lack of niches further, it can potentially trigger secondary extinctions, especially if $d = 0$ before the removal of the species. If the removal of a species causes d to decrease (which is possible only if $d > 0$ before the removal), then the removal of the species could potentially make the food web more compatible with coexistence. It should however be noted that — especially in food webs containing

parasites — removal of species, even when decreasing the nullity, can lead to strong disruptions. E.g., when parasites use different hosts at distinct life stages (Lafferty et al., 2008), removal of only one of the hosts could be sufficient to cause extinction of the parasite.

We tested how removal of each species affects d in the seven food web data sets. We again distinguish food webs with free-living species only, with parasites, and with parasites and concomitant links. For simplicity, symmetric entry to the matrix is assumed for concomitant links. In Fig. L, species are labeled based on their average chain length. Species for which removal increases d are marked by red symbols ("+"), those for which removal decreases d are marked by green symbols ("×").

For the free-living species webs with $d > 0$ (Bahia Falsa, Flensburg Fjord, Otago Harbor, Sylt Tidal Basin, and Ythan Estuary), there is a clear tendency that removal of species with chain lengths near one or three increases d, while the removal of species near the chain length two or four decreases d. This is consistent with the observation that the excess species at the even trophic levels cause the lack of niches (cf. Fig. M).

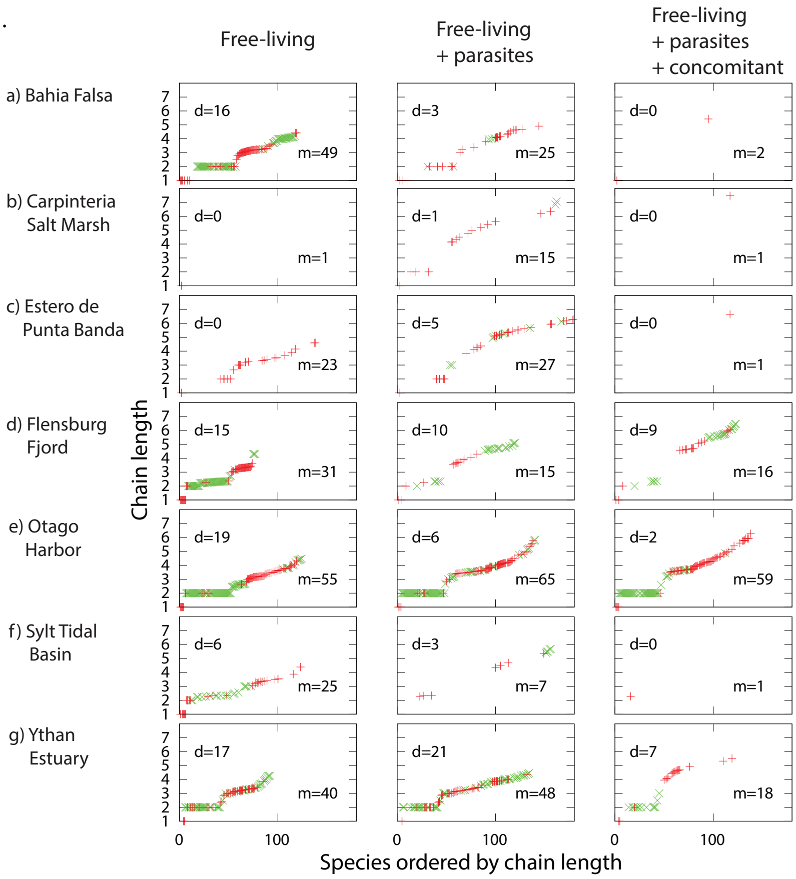

Figure L: Effect of removal of a species on the lack of niches d. The species are labeled based on their average chain length. If the removal of a species results in increase of d, the species is marked by a red + (potentially triggering the secondary extinction), while if the removal of a species results in the decrease of d, the species is marked a green ×. If d does not change, no symbol is given. In the figure, the value of d before removal is given. Number of species, m, that increase d is also given in each figure.

For the webs with $d = 0$ before removal (Estero de Punta Banda, Carpinteria Salt Marsh), only an increase of d can occur, and removal of such species will cause secondary extinction. The Estero de Punta

Banda free-living web is quite sensitive to the removal of one species, while CA is not.

When considering food webs including parasites, it must again be noted that substantial disruption (and extinction) could be caused, when only one of the parasites' hosts' is removed. Given this limitation, we state for Bahia Falsa, Flensburg Fjord, and Sylt Tidal Basin, that adding parasites could in principle reduce the sensitivity of the food web to the removal, as seen by reduced values of the number of species m that increase d by removal. Carpinteria Salt Marsh and Punta Banda appear to be less stable when only adding parasites since $d = 0$ was already realized in the free-living species web, but concomitant links might have the potential to stabilize these webs. Ythan Estuary also shows such a tendency, though it starts with a rather high value of d in the free-living species web.

Otago Harbor remains rather sensitive to the removal of species even with addition of parasites. This may be due to the rather large excess number of species in the trophic level 2 (cf. Figure M). The addition of parasites decreases d but not enough to completely compensate the excess species, which make it sensitive to removal of species in other levels and also removal of species from level 2 helping to reduce d for all the three cases.

S10 Modeling

This section provides modeling of an idealized food web structure. Sec. S10.1 states the basic assumptions made to obtain an idealized, food web with sharp trophic levels (no omnivores). Originating from this "free-living" food web, we discuss several perturbations: Modification of the free-living food web to include omnivores (Sec. S10.2), addition of different types of parasites to the free-living food web (Sec. S10.3), as well as a discussion of asymmetric and symmetric concomitant links (Sec. S10.4).

S10.1 Idealized food web structure

To obtain additional insight into the generic features of the data, we now produce an idealized free-living food web. To this end, we consider all seven empirical food webs and determine the species' approximate trophic levels (Fig. M.a and b). For simplicity, for any species we assign the closest integer trophic level as its default trophic position, e.g. a species with mean chain length 3.2 would be assigned a value of 3. Proceeding in this way for all food webs, indeed some degree of generality is found (compare Fig. M.a and b): Basal species constitute the smallest fraction of trophic levels, levels 2 and 3 are most diverse and level 4 often constitutes the top level (for details, see also the table in Fig. M.c). Four webs have a small number of species (between 1 and 3) at level 5, which we ignore for simplicity. We thereby arrive at a generic distribution of species richness in terms of trophic levels. This particular distribution is characterized by an inherent lack of niches: a default nullity of 22 is predicted since $N_o - N_e = -22$ (compare Sec. S5). In terms of resource vs. consumer limitation (Sec. S3) the system would be in a state where far too many consumers were added to be consistent with the assembly rules (we call this *consumer-dominated* in the following). We note that a compensation by nutrient sources is thereby not possible, since additional nutrient sources could only be used to compensate for an "overweight" in terms of unpaired basal species. In the current situation, however, compensation would be required for species at levels 2 or 4.

By inspecting the table in Fig. Mc, we further find that in each of the empirical food webs, the difference $N_o - N_e$ is negative, indicating that all webs are in a consumer-dominated state, an inherent nullity, or rank deficiency, hence results for all food webs, ranging from -9 for the Ythan Estuary web to -37 for Otago Harbor. This means, if the food webs were organized strictly in terms of these integer trophic positions (no variation of food chain length for a given species), the assembly rules could not be fulfilled for any of them. One can speculate as to the origin of the imbalance in $N_o - N_e$. One explanation could be that basal species are poorly resolved. Another is that parasites can be seen as adding a counterweight at higher trophic levels and cause additional mixing.

Using the idealized structure as a starting point, we initially assume strict trophic positions of all species and assign random interactions within the allowed sub-block of the matrix until the empirical

a

b

e

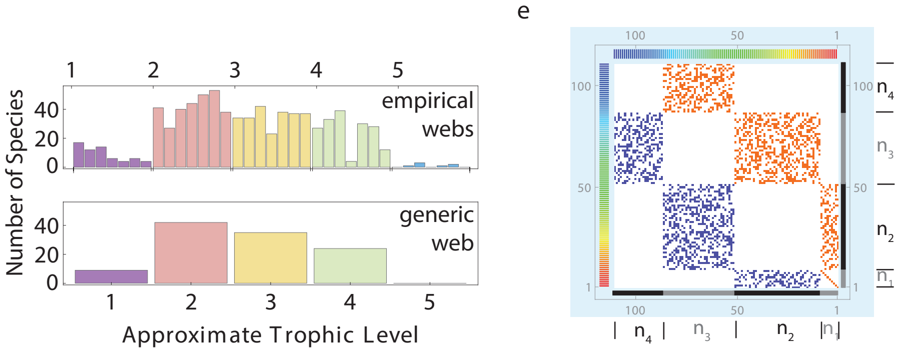

c

Data set	n_1	n_2	n_3	n_4	n_5	N_o-N_e	S_f	S_p	S_p/S_f
Bahia Falsa	17	41	34	27	0	- 17	119	52	0.44
Carpinteria	12	27	34	33	1	- 13	107	58	0.54
Punta Banda	14	40	42	39	3	- 20	138	76	0.55
Flensburg Fjord	6	44	23	4	0	- 19	77	46	0.60
Otago Harbor	4	50	38	30	1	- 37	123	19	0.15
Sylt Tidal Basin	6	53	37	28	2	- 36	126	35	0.28
Ythan Estuary	4	38	37	12	0	- 9	91	42	0.46
**Idealized data**	**9**	**42**	**35**	**24**	**0**	**- 22**	**110**	**47**	**0.43**

d

Data set	L_f	L_p	$L_{p\text{-}p}/L_p$	L_p/L_f	L_p/S_p	d_f	d_{f+p}	$d_{c,asym}$	$d_{c,sym}$
Bahia Falsa	1077	1157	0.14	1. 07	22.2	16	3	3	0
Carpinteria	970	1217	0.14	1. 26	21.0	0	1	1	0
Punta Banda	1657	1677	0.10	1. 01	22.1	0	5	5	0
Flensburg Fjord	579	389	0.10	0. 67	8.5	15	10	10	9
Otago Harbor	1206	281	0.07	0. 23	14.8	19	6	4	2
Sylt Tidal Basin	1052	898	0.08	0. 85	25.7	6	3	3	0
Ythan Estuary	420	177	0	0. 42	4.2	17	21	12	7
**Idealized data**	994	←				**varies**			→

Figure M: **Idealized food web data for simulations.** **a**, Approximate species richnesses n_i at trophic levels: Approximate trophic positions were determined by rounding the species chain lengths to the nearest integer value. For similar colors, bars from left to right correspond to the sequence of food webs given in the table (c). **b**, Generic web used in the simulations: Values of n_i were obtained by averaging the corresponding trophic richnesses of species in the empirical food webs and rounding to the nearest integer value. **c**, Table of food web data for all empirical and the idealized food web. n_i are the numbers of species at trophic levels i obtained by the rounded mean food chain lengths for all species in the respective webs. $N_o - N_e$ is the difference of the numbers of species at odd and even levels, respectively. S_f and S_p are the numbers of free-living and parasite species, respectively. **d**, Table containing information on links in the different food webs: L_f are the numbers of links in the free-living web, L_p are the numbers involving at least one parasite. L_{p-p} are the numbers of links between parasites. d_f and d_{f+p} denote the nullities in the webs of free-living, respectively free-living and parasite species webs. $d_{c,asym}$ and $d_{c,sym}$ denote the nullities when including asymmetric and symmetric concomitant links, respectively. The table also indicates where values vary in the simulations. **e**, An example of a sample of the idealized free-living food web matrix $\mathcal{R}^{sim}_{free}$, used as a starting point for the simulations: All species are placed at integer-valued trophic positions without food chain variation. $\{n_1,n_2,n_3,n_4\}=\{9, 42, 35, 24\}$. Basal species are all assigned individual nutrient sources (yielding a sequence of diagonal elements in the lower right block matrix). Panels (a), (b) and (e) are a reproduction of main text Fig. 6a,b.

average of links is reached (compare table Fig. Md). The strict trophic positions require that the distribution of chain length variation would be a spike at zero. In the following, we refer to this type of matrix as $\mathcal{R}^{sim}_{free}$, a sample is shown in Fig. Me. In sections S10.2 and S10.3 we use $\mathcal{R}^{sim}_{free}$ as a starting point for the discussion of two specific types of modifications: additions of omnivores to $\mathcal{R}^{sim}_{free}$ by re-assignment of links (no link addition); addition of parasites to $\mathcal{R}^{sim}_{free}$ (increasing total number of species to $S_f + S_p$, see Fig. Mc).

S10.2 Simulations of omnivore addition within the free-living web

We apply two types of perturbations to $\mathcal{R}^{sim}_{free}$ without changing the number of species or links:

- Some of the links described by $\mathcal{R}^{sim}_{free}$ are reassigned to become omnivorous links (Fig. N.d). Even for a small fraction of omnivorous links, the variation of chain lengths widens and at 65 links already becomes similar to that of the aggregated empirical data (compare Fig. N.a). Addition of omnivorous links is further associated with a rapid reduction of the nullity (Fig. N.f, dashed line).
- We consider specific omnivory, namely only that of level 3 species (which initially feed only on level 2) being able to feed on other level 3 species (3-3 interaction in addition to the existing 3-2 interaction). This type of interaction appears to be quite abundant in the empirical data (Fig. U). In this case, the distribution of chain length variation changes more slowly, even at 125 omnivorous links the empirical distribution has not yet been attained. Considering again the change of nullity as function of added links, there is no change. This is clear as the addition of 3-3 interactions does not help in relaxing the consumer-dominated state of the systems towards a more balanced configuration. In terms of the species pairing, 3-3 interaction would allow for pairing of level 3 species with level 3 species. However, all existing level 3 species were previously already paired with either level 2 or 4. Conversely, more 4-2 or 2-2 interactions would be beneficial, as in that case e.g. the abundant level 2 species could be paired with other species at that level or with some of those at level 4.

Real food webs do have high density of 3-3 interactions but some also show substantial 4-2 interaction. Actual food webs therefore likely lie in between the two extremes (solid and dashed lines in Fig. Nf). However, nearly no 2-2 interactions are present in empirical webs, which makes pairing between such species rare.

S10.3 Simulations of parasite addition

We now perform a similar analysis for webs including parasites (Fig. O). Starting again from the idealized free-living food web $\mathcal{R}^{sim}_{free}$ (Fig. O.a), we perform four distinct simulations:

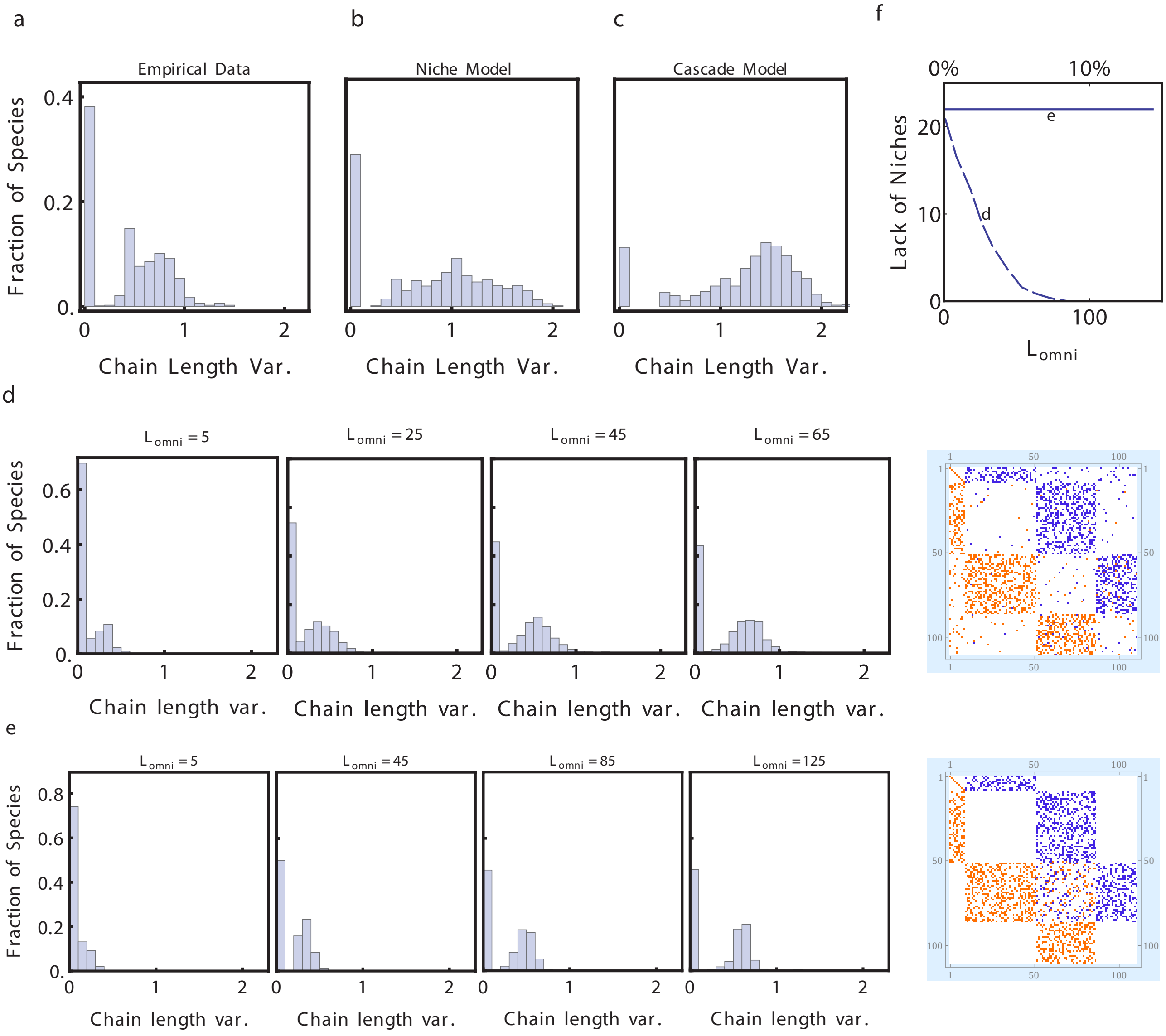

Figure N: **Chain length variation for free living species.** Normalized histograms of the standard deviation of food chain length for free living species food webs. **a**, Aggregated empirical data for all seven empirical food web data sets. **b**, Niche model results for corresponding values of S and L for the seven food webs. **c**, Similar to (b) but for cascade model results. **d**, Simulation data for the idealized free-living food web containing the average numbers of species at approximate trophic levels 1, 2, 3, and 4. The corresponding node richness equals $\{9, 42, 35, 24\}$, respectively, leading to a theoretical lack of niches of 22. For $L_{omni} = 0$ each species in the constructed food web only consumes species belonging to the trophic level below, i.e. all species have zero food chain length variation. Panels show results of simulation using varying numbers of omnivorous links (L_{omni}, as labeled) to perturb a system of species with well-defined trophic levels. Omnivorous links produce consumer-resource pairs where the difference in trophic levels between the respective partners is larger than 1. **e**, Similar to (d) but for omnivorous links only allowed between species formerly located at trophic level three and constraining the omnivorous links to include interaction with another species at level three. **f**, Dependence of rank difference ("lack of niches") on L_{omni} for the two cases shown in d and e as dashed and solid curves, respectively. Percentages on top horizontal axis specify the fraction L_{omni}/L, i.e. the fraction of omnivorous links.

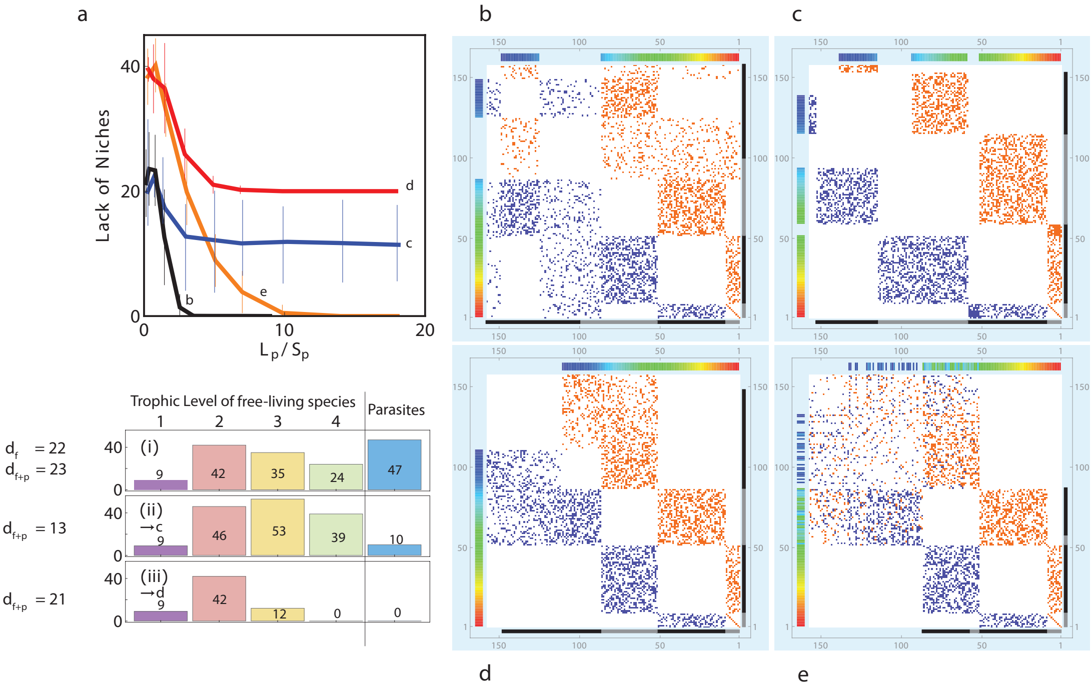

Figure O: **Simulations of different food web matrices.** **a**, The lack of niches d as a function of the number of links per parasite for the different cases b—e (as labeled). Vertical lines indicate the standard deviation of results when repeating for many different samples of food web matrices. Barplot indicates the resulting distributions of node richness on the respective trophic levels when considering parasites as an additional trophic level (i), in the case described in **c**, (ii), as well as that described in **d**, (iii). **b**, Addition of parasites that form random links to existing free-living species. **c**, Addition of parasites that are confined to consumption on a specific trophic level. **d**, Similar to (b) but with the restriction of parasites consuming only free-living species at levels 3 and 4. **e**, Similar to (d) but with additional parasite-parasite interactions (hyperparasitism), approximately 5 percent of parasite links are from parasite to parasite. Panels are reproduced from main text Fig. 6.

1. Addition of the empirical number of parasite species that interact with any existing levels at random, to ensure that each parasite will be connected. Initially, each parasite is granted one link to a random existing species. All subsequent links are completely random (Fig. O.b).

2. Addition of parasites that are specialized at consuming species of a given trophic level. All parasites are thereby first assigned a random host, this defines the specific trophic level of hosts for the given parasite. Subsequent links are then added by ensuring that the given parasite will only consume hosts with identical chain lengths to the first. Link additions are otherwise random (Fig. O.c).

3. Initially, each parasite is given one link to an existing free-living species at level 3 or 4. Subsequent links are assigned randomly between any parasite and any level 3 or 4 species (Fig. O.d).

4. Similar to (d) but parasites consume hosts at levels 3 and 4 and also previously added parasites at random (Fig. O.e). Approximately a 5 percent fraction of parasite links are parasite-parasite links.

Simulations show qualitatively different results for the four cases: (1) Random addition and consumption behavior of parasites leads to rapid degradation of the nullity, addition of less than 5 links per parasite generally suffices to remove the nullity, i.e. allow full rank. (2) Completely level-specific addition of parasites leads to a slight initial reduction of rank. This is due to a compensating effect of adding parasites to random hosts: Dominant trophic levels will acquire more parasites. Those parasites are then counted towards the node richness at the neighboring (higher) trophic level. Apart from this (statistical) effect, the addition of level-specific parasites can not remove the nullity. (compare the barplot in Fig. O.a,(ii) where we also indicate the expected nullity when adding parasites). (3) A similar effect is initially seen for parasites that consume at two trophic levels. There, an initial increase in nullity is found due to the larger likelihood of consuming at level 3, thus adding more species to level 4 and thereby increasing the consumer-dominated state. The nullity however declines when further links are added and saturates at a value of $d_{f+p} = 21$, which is obtained by pairing parasites with all level-4 species and the remaining unpaired parasites with level 3 (compare Fig. O.a,(iii)). Another case arises, when parasites are viewed simply as adding an additional trophic level, i.e. all parasites consume exclusively level-4 species. This case in fact yields again a completely structured food web, where simply a level 5 with $n_5 = 47$ species is attached to the existing free-living web. The assembly rules then predict that only $n_4 = 24$ parasites can be paired, leaving $n_5 - n_4 = 23$ parasite species unpaired. This sets the nullity of the resulting matrix (Fig. O.a,(i)). This also implies that adding parasites can in fact increase the nullity, if hosts are dominantly located at a specific, single trophic level. (4) When parasites can also consume other parasites (in the empirical data typically more than five percent of parasite links involve parasite-parasite interaction) the nullity can again be removed and approximately 10 links per parasite are sufficient for the moderate concentration of parasite-parasite links used here.

Within the limitations of the idealization made, reality is best described by the curve corresponding to (Fig. O.e), when also the order of interaction is random, i.e. in some cases parasites consume free-living species, but in others the order is reversed. This best mimics the overall features seen in the data (Fig. U), also regarding the shuffling of average chain lengths (indicated at the matrix boundaries in color shades). Note also, that in real food webs there are also interactions between parasites and level-1 and level-2 species (Figs U and J), which could also be included in the model.

Specific food webs (details). We again compare with several empirical cases:

- The Bahia Falsa web (Fig. U.a) might be reasonably well described by Fig. O.e with substantial interaction between parasites and level-3 and level-4 free-living species but also some interaction within the parasite-parasite sub web. This may explain the substantial reduction of nullity for that web ($d_f = 16 \rightarrow d_{f+p} = 3$, compare Fig. M.c,d).

- The Ythan Estuary food web (Fig. U.g) does not include any parasite-parasite interaction and shows relatively strict hierarchical organization, even for interactions involving parasites. Hence,

that particular web might be best described by Fig. O.d, leading to a sizable nullity, even with parasites ($d_f = 17 \rightarrow d_{f+p} = 22$). We have tested, whether adding several random parasite-parasite interaction to that food web could change the nullity. We find that when adding on average one parasite-parasite link to each existing parasite (the approximate value of the other food webs), the nullity decreased to values as low as 7 or 8.

- Ythan Estuary further has comparably low numbers of links per parasite ($L_p/S_p = 4.2$ compared to an overall average of more than 15), which further makes it difficult to achieve full rank.
- Another food web, Flensburg Fjord, maintains relatively high nullity ($d_{f+p} = 10$). There, adding further random parasite-parasite interactions can even reduce the nullity to zero.
- Otago Harbor stands out with a very small fraction of parasite species ($S_p/S_f = 0.15$, compared to an average of 0.43), but an overweight of even-level species of 37. This extreme configuration in the measured data might possibly be a hint of an under-sampling of parasite species in addition to the possible under-sampling of basal species.

S10.4 Simulations of the effect of concomitant predation

We also perform simulations to describe the effect of concomitant predation. There are two qualitatively different effects present in the empirical data (Fig. Nd):

1. Symmetric concomitant links lead to an overall improvement of rank, i. e. a reduction in rank deficiency.
2. Asymmetric concomitant links (only affecting the parasite) lead to an improvement in rank in only few cases, most food webs do not show any change in rank.

To start the discussion, it is important to note several empirical observations: Parasites predominantly interact with species at more than one trophic level, e.g. as implied by the variation in their chain lengths (Fig. AA). A substantial fraction of free-living species do not have parasite interactions (table in Fig. J), e.g. approximately 50 percent for the Ythan Estuary web. In many webs, parasites also interact with other parasites (Fig. Mc,d), but there are exceptions (Ythan Estuary).

Parasite-free-living links only. To allow for a simple model, we again start from the idealized food web structure (Fig. M.b) and the matrix $\mathcal{R}^{sim}_{free}$. We then include a typical number of parasites (set to 47), and allow these to prey on a fraction (we choose 50 percent for simplicity) of species at levels 3 and 4 with otherwise random links (Fig. P.a). In practice, parasites do prey on all trophic levels (albeit with some bias towards the higher levels). Our theory can easily be extended to more levels.

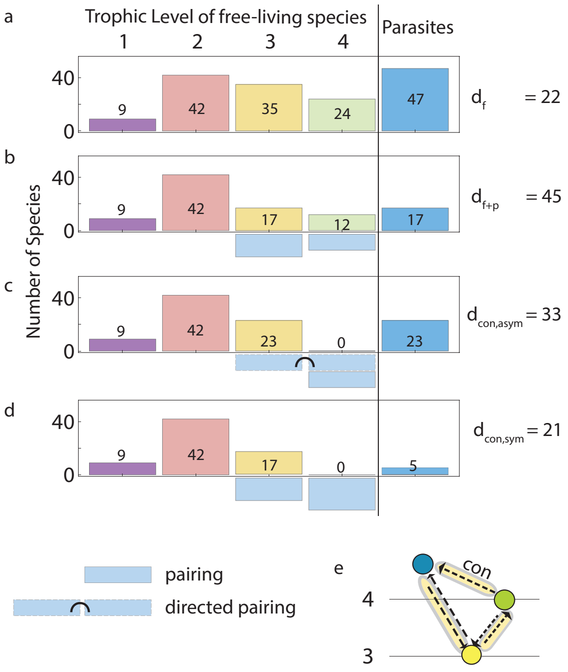

Figure P: **Schematic of trophic levels with concomitant predation.** **a**, Free-living food web. **b**, Inclusion of parasites feeding on a subset of hosts at trophic levels 3 and 4. After pairing, there are 17 remaining "unpairable" parasites, n_3 and n_4 have been reduced to 17 and 12 unpaired species, respectively. **c**, Inclusion of asymmetric concomitant links in the web shown in (b). Use of free-living species for pairing indicated by blue boxes on negative vertical axis. Use of concomitant links for directed pairing indicated by dashed blue boxes on negative vertical axis. **d**, Including also symmetric concomitant links. **e**, Schematic showing the type of concomitant links present in the simulated food web. "con" indicates the concomitant link. Numbers indicate the trophic levels of the parasite's (blue) free-living host (yellow) and host's predator (green). Panels also show the respective niche deficiencies d_f, d_{f+p}, $d_{con,asym}$, and $d_{con,sym}$.

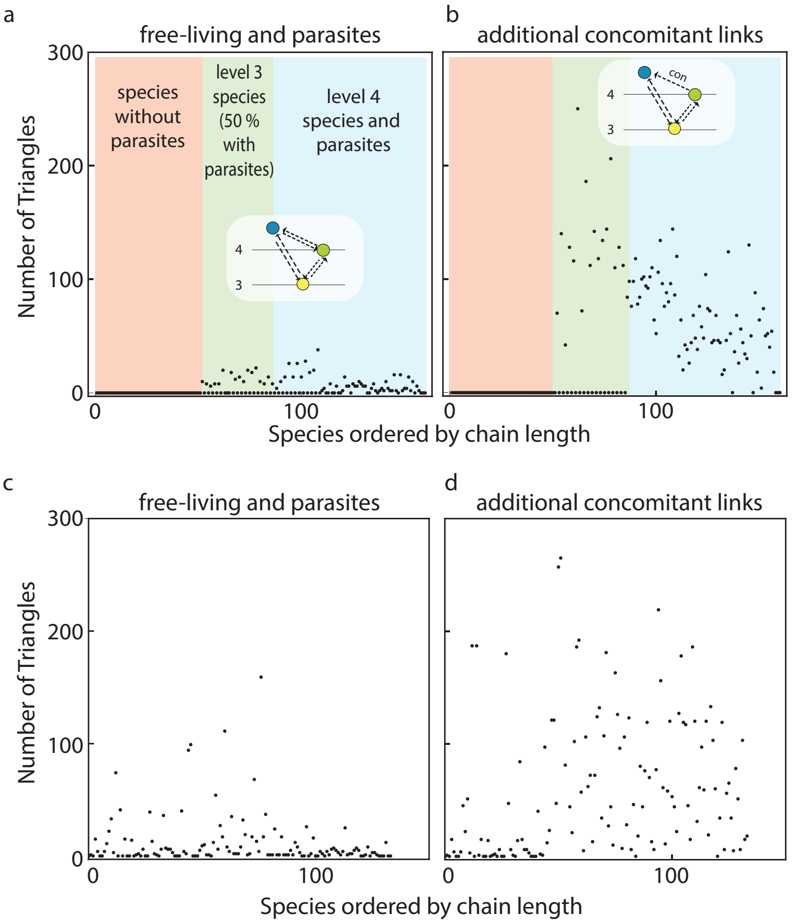

Figure Q: **Numbers of loops of length three for simulations and empirical data.** **a**, Food web formed by free-living and parasite links. Schematic in (a) indicates the types of triangles that can be formed (bi-directional between level 3, level 4 and parasite species, shown in yellow, green and blue, respectively); **b**, Food web including additional concomitant links. Schematic in (b) indicates the additional type of triangle that can be formed when including concomitant links. **c**, as in (a) but for the empirical Ythan Estuary data (reproduction of Fig. Jg, middle panel). **d**, as in (c) but for the empirical Ythan Estuary data (reproduction of part of Fig. Jg, right panel).

Given a sufficient number of links, the usual pairing will allow those free-living species which interact with parasites to pair with these, i.e. half the species at levels 3 and 4 can pair with parasites, leaving a reduced number of unpaired (and "unpairable") parasites (see panel b). Collecting the remaining unpaired free-living and parasite species, the lowest possible nullity is $d_{f+p} = 45$.

Asymmetric concomitant links. We now examine how inclusion of asymmetric concomitant links can improve the situation. Consider the directed loop shown in Fig. Pe and let all level-4 species, previously lacking connections to parasites, participate in such loops of length three (this requires sufficient numbers of links, but as we will see in the simulations, this is usually possible). The loops engage $n_4/2$ triples in directed pairings. The remaining $n_4/2$ free-living species are open to bi-directional interactions with parasites and can hence also be paired. We arrive at the situation shown in Fig. Pc, where the types of pairings are indicated. Notably, the nullity has now been reduced to $d_{con,asym} = 33$.

Symmetric concomitant links. Consider now symmetric concomitant links (Fig. Pd). In this case, level-4 free-living species are likely to acquire bi-directional links to parasites and can all be paired. Level-3 species however do not acquire additional links to parasites, only half of these could be paired. In the example at hand, there is already an overweight of even level species, level-3 species can therefore be

equally well paired with level-2 species. In any case, the nullity can now be decreased to $n_{con,sym} = 21$.

We have simulated all the above cases using the idealized matrix $\mathcal{R}_{sim}^{free}$ (Fig.R.a—c). In particular, we have also checked if indeed more loops of length three are produced when adding concomitant links. Fig. Q shows the number of triangles when simulating free-living and parasite species alone (shown in panel a), as well as additional concomitant links (panel b). In the panels, we distinguish three groups of species: those without parasites (levels 1 and 2), those at level 3 (some have parasites) and those at level 4 or parasites. Notably, in Fig. Q.a, loops of length three can only be formed when a parasite interacts both with a species at level 3 and level 4 and additionally, the level 4 species preys on the level 3 species (shown in the schematic). This means, that any free-living species, that does not directly interact with a parasite, can not be involved in a loop of length three (there are correspondingly a number of species without loops, even in levels 3 and above). Conversely, when adding concomitant links, additional triangles can be formed (see the schematic in Fig. Q.b). Notably, level 4 species that were not involved in any triangles in Fig. Q.a now often have substantial numbers of triangles. These idealized simulation results should be compared to Fig. J.g, central and right panels (Ythan Estuary web, reproduced also in Fig. Q.c,d). Many of the features are similar, in particular, the increase of the number of triangles when adding concomitant links, the large number of free-living species without triangles in the case without concomitant links, as well as the reasonable quantitative agreement. The plots differ in that the actual data does have some triangles for low-level species, which is due to the fact that parasites do interact with some species at those levels as well, and omnivory does exist for free-living species.

Additional parasite-parasite links. When a small fraction of links between parasites are included, the system will acquire the ability to pair many parasites with other parasites, which can sometimes allow all parasites to be paired with others. However, a subset of parasites might be needed to pair free-living species, in the example, a number of level-4 species should be paired to reduce the overweight of the even levels. As only $n_4/2$ of these have parasite links, only these can be paired. The nullity can then be reduced to $d_{f+p} = 10$. Adding additional asymmetric concomitant links will not help, since those always involve both a level-3 and a level-4 species. In this case, there will be no benefit in such 3-species pairs, since they will not reduce the overweight of the even levels. The situation is different for symmetric concomitant links, where additional pairings between level-4 species and parasites become possible, thereby reducing the overweight of the even levels. Simulating the case where approximately 15 percent of parasite links were parasite-parasite links, overall reduction of the nullity is achieved (Fig.Rd), even without inclusion of concomitant links. However, considering now the inclusion of asymmetric concomitant links (Fig.Re), the nullity is not decreased further ($d_{f+p} = f_{con,asym} = 10$). When including symmetric concomitant links, full rank is achieved. This type of simulation (where some parasite-parasite links are present) might be a reasonable abstraction of what is seen in the webs in Fig. U.a,b,c,d,f, where the nullity changes with symmetric, but not with asymmetric, concomitant links.

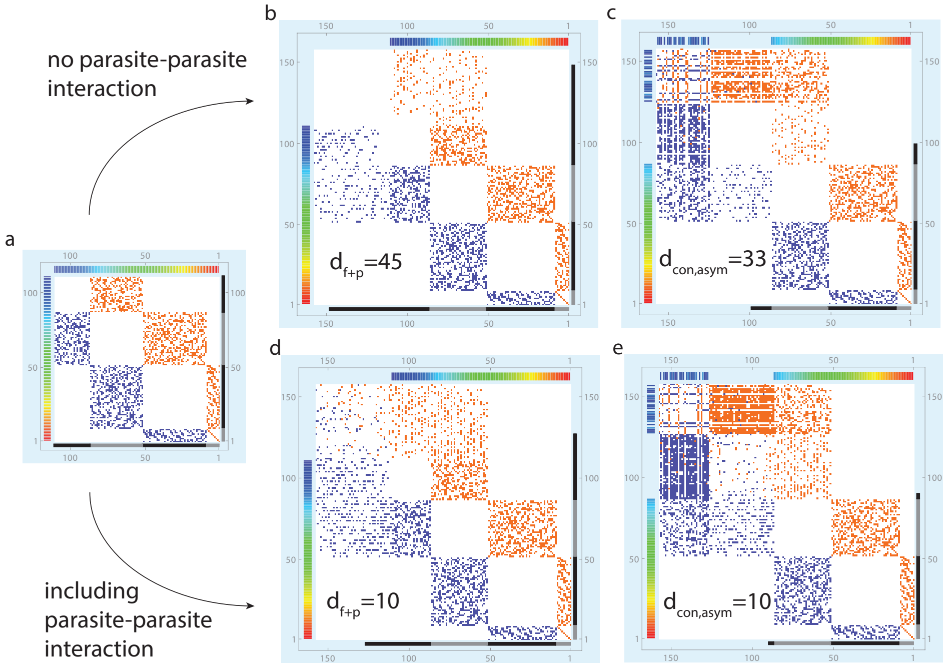

Figure R: **Food web matrices for simulated concomitant predation.** **a**, Free-living food web (reproduction from Fig. O). **b**, Inclusion of parasites feeding on a subset of hosts at trophic levels 3 and 4. **c**, Inclusion of concomitant links in the web shown in (b). **d**, As (b) but including some parasite-parasite interaction. **e**, Inclusion of concomitant links in the web shown in (d).

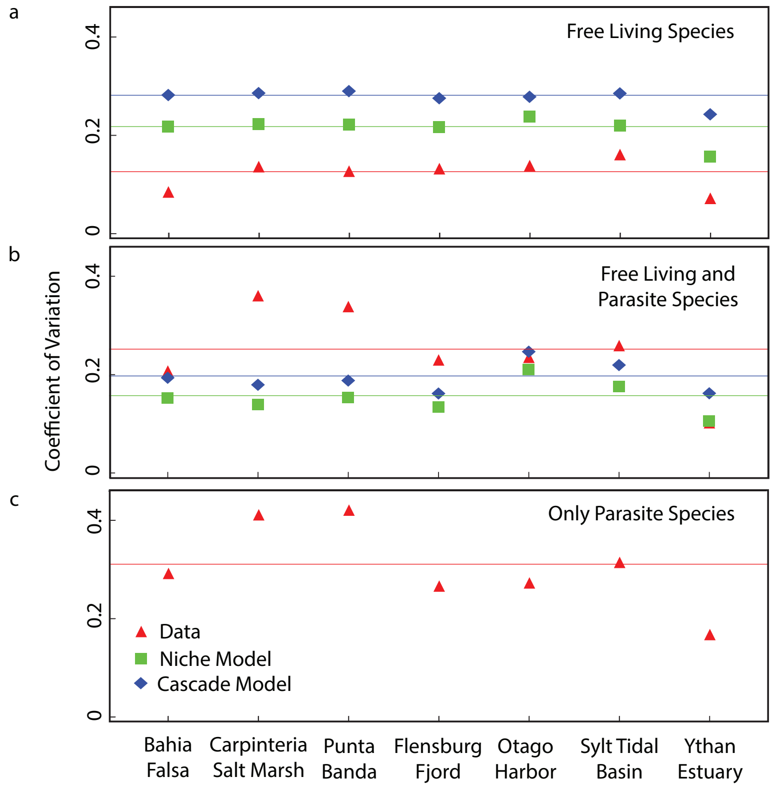

Figure S: **Coefficient of variation for chain lengths of species.** **a**, Food web with consisting of free living species only. **b**, Food web consisting of free living and parasite species. **c**, Similar to (b) but chain length variation coefficient for chains originating at parasite species species only.

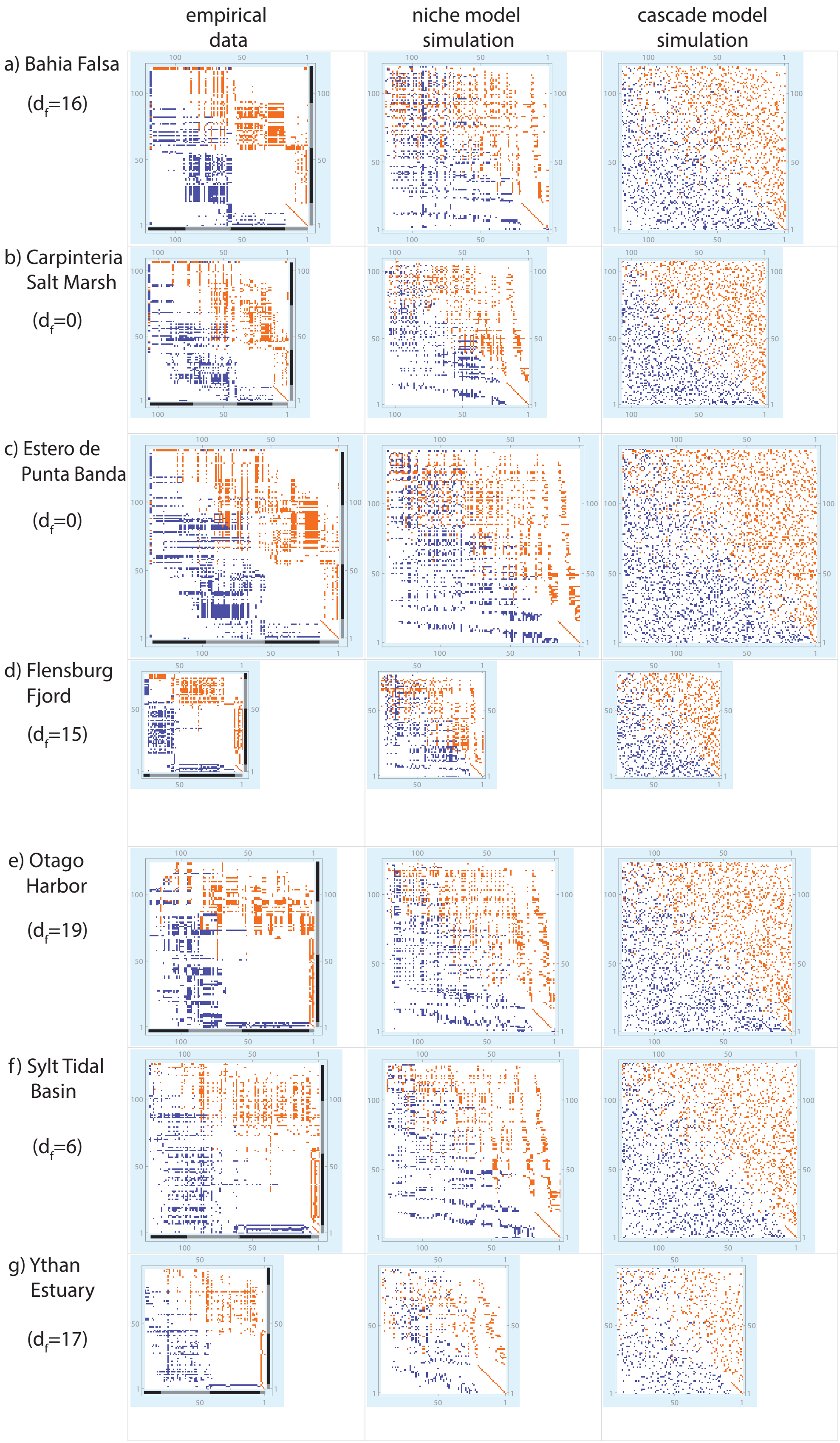

Figure T: **Free living food web matrices compared to niche and cascade models. a—g**, Food webs as labeled. Empirical data (left column), corresponding niche model simulation (central column) and cascade model simulation (right column). Matrix indices organized by approximate food chain length (basal species: bottom right). Blue and red matrix elements indicate direction of links. Black and gray stripes at boundary of empirical food web matrices indicate ranges of approximate trophic levels.

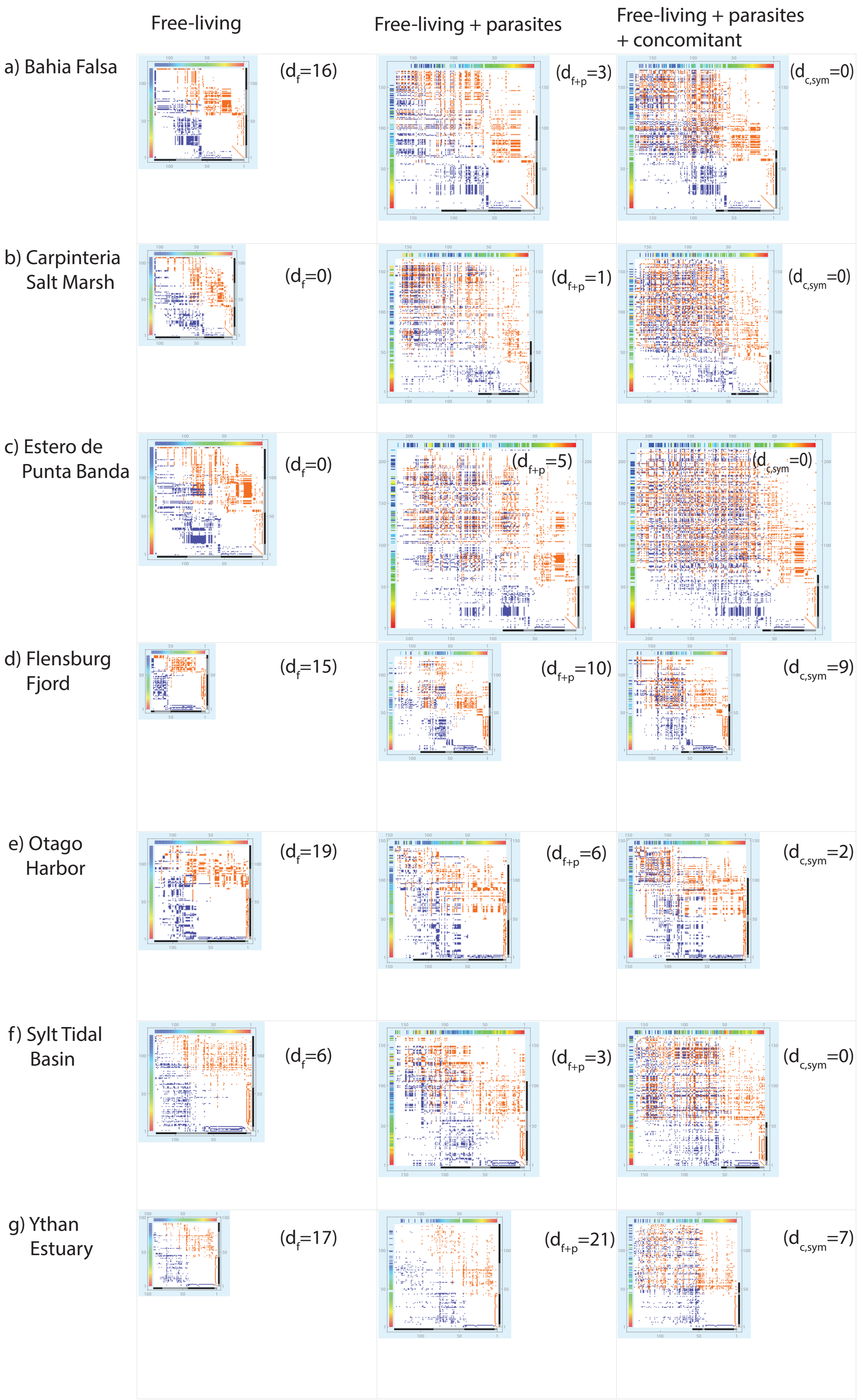

Figure U: **Empirical food web matrices. a—g**, Food webs as labeled. Empirical data for free-living webs (left column), free-living and parasite species webs (central column), as well as those including additional concomitant links (right column). Matrix indices organized by approximate food chain length (basal species: bottom right). Blue and red matrix elements indicate direction of links. Black and gray stripes at boundary of empirical food web matrices indicate ranges of approximate trophic levels. Additional color-coding along the matrix boundaries indicates the respective location of species in free-living webs in the parasite webs, e.g. most basal and level-2 species remain at approximately unchanged locations.

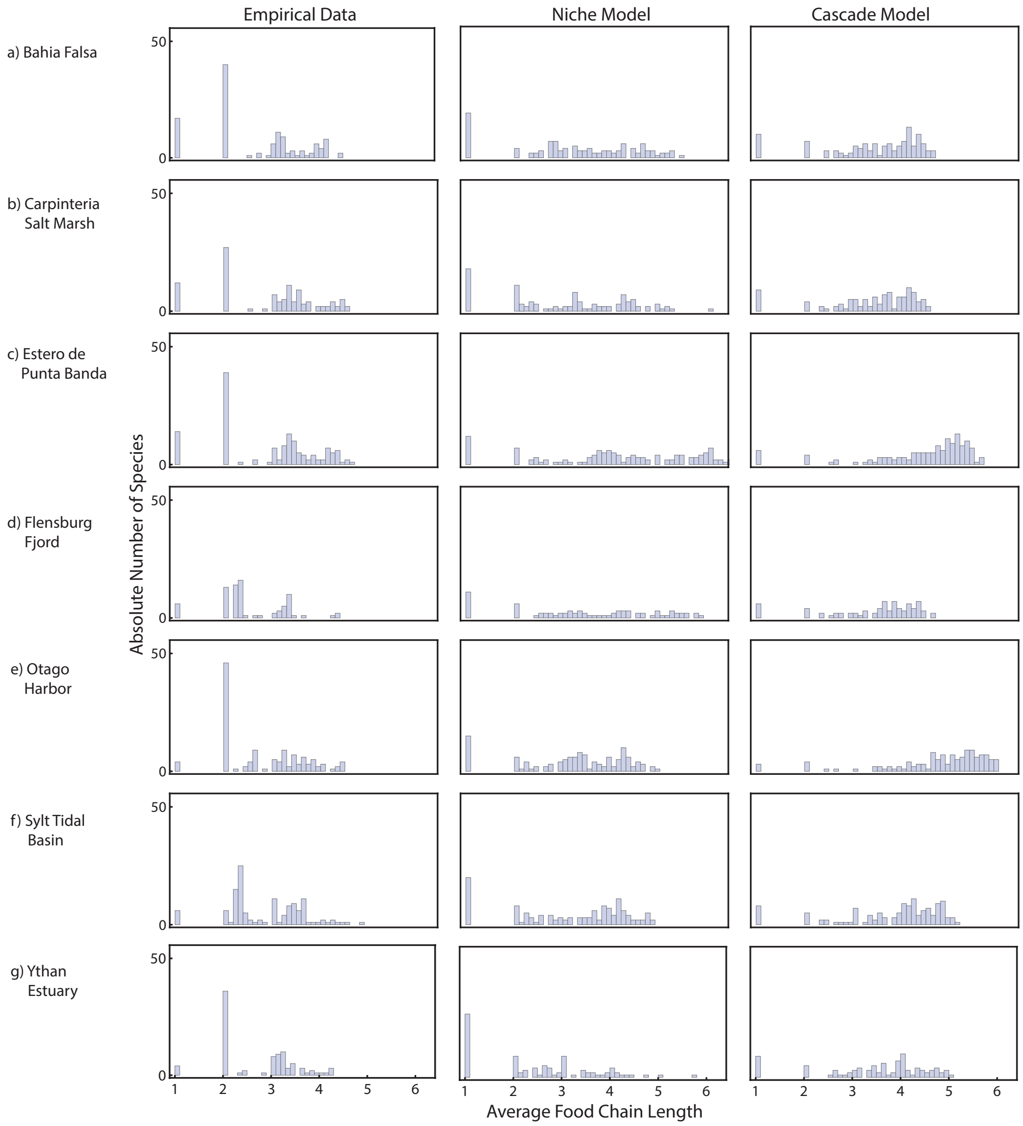

Figure V: **Chain length distributions for free living species.** Average food chain length distribution for free living species food webs. Empirical data/niche model/cascade model shown in left/middle/right columns. **a—g** denote the seven food webs Bahia Falsa, Carpinteria Salt Marsh, Estero de Punta Banda, Flensburg Flord, Otago Harbor, Sylt Tidal Basin, and Ythan Estuary, respectively.

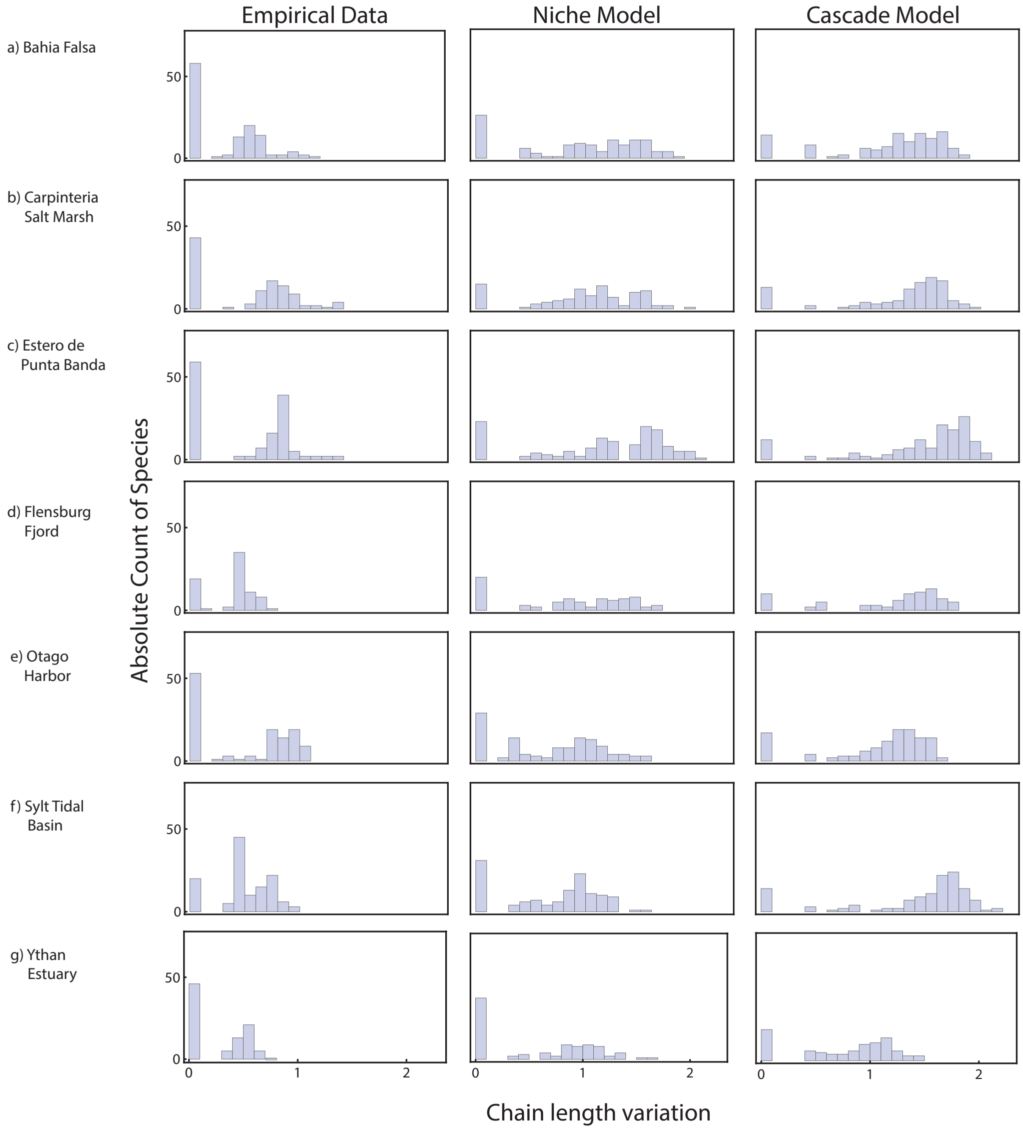

Figure W: **Food chain length variation for individual food webs.** Histograms of species with a given value of food chain length variation. **a—g** denote the seven food webs Bahia Falsa, Carpinteria Salt Marsh, Estero de Punta Banda, Flensburg Flord, Otago Harbor, Sylt Tidal Basin, and Ythan Estuary, respectively.

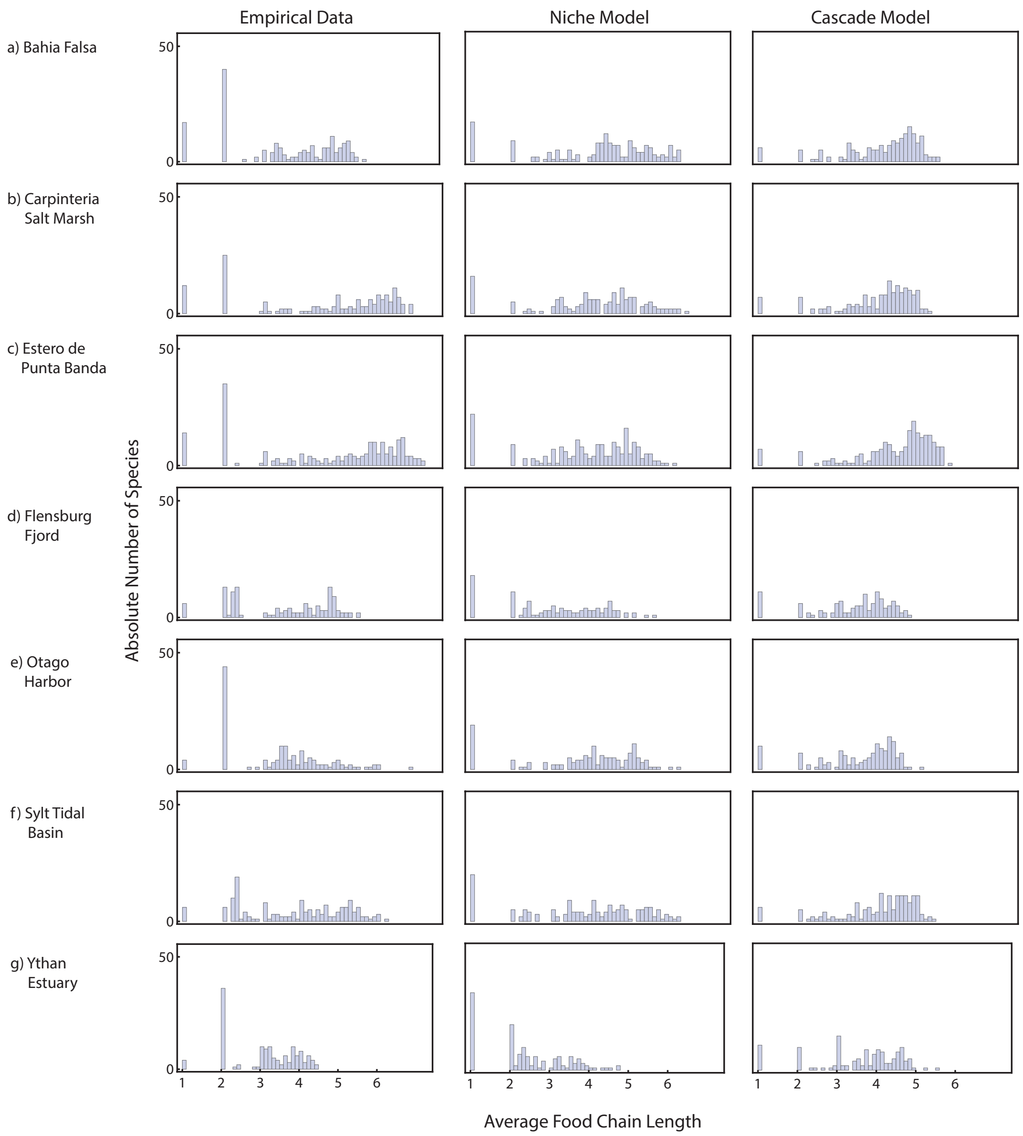

Figure X: **Chain length distributions for food webs including parasite species.** Similar to Fig. V but for webs including free-living and parasite species.

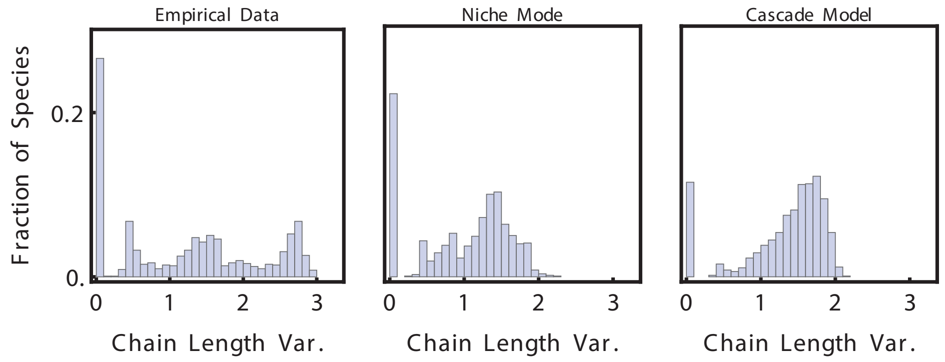

Figure Y: **Chain length variation including parasite species (aggregated data).** Normalized histograms of the standard deviation of food chain length for food webs containing free living and parasite species. **a**, Aggregated empirical data for all seven empirical food web data sets. **b**, Niche model results for corresponding values of S and L for the seven food webs. **c**, Similar to (b) but for cascade model results.

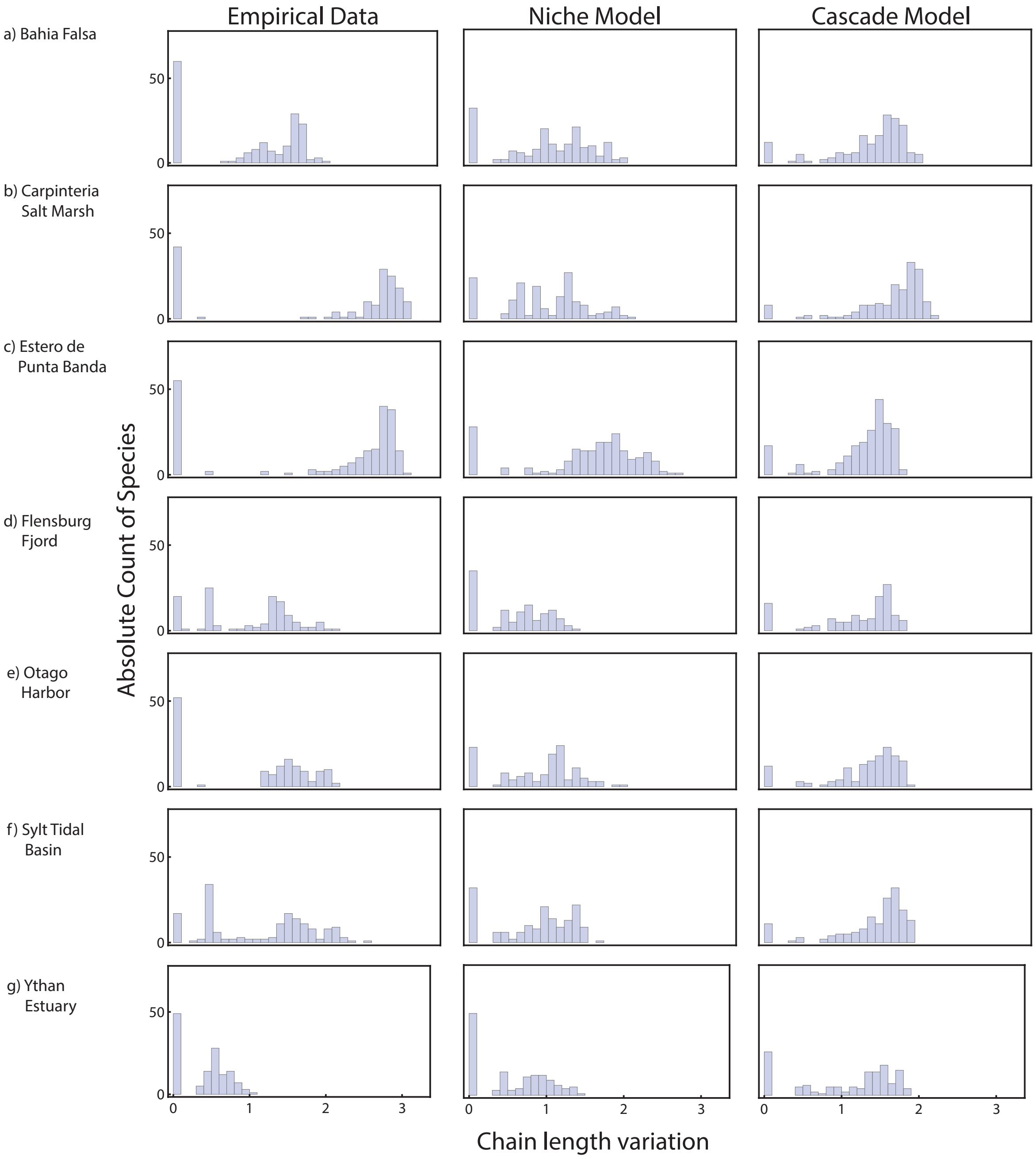

Figure Z: **Chain length standard deviation including parasite species.** Standard deviation of food chain length for free living and parasite species food webs. Empirical data/niche model/cascade model shown in left/middle/right columns. **a—g** denote the seven food webs Bahia Falsa, Carpinteria Salt Marsh, Estero de Punta Banda, Flensburg Flord, Otago Harbor, Sylt Tidal Basin, and Ythan Estuary, respectively.

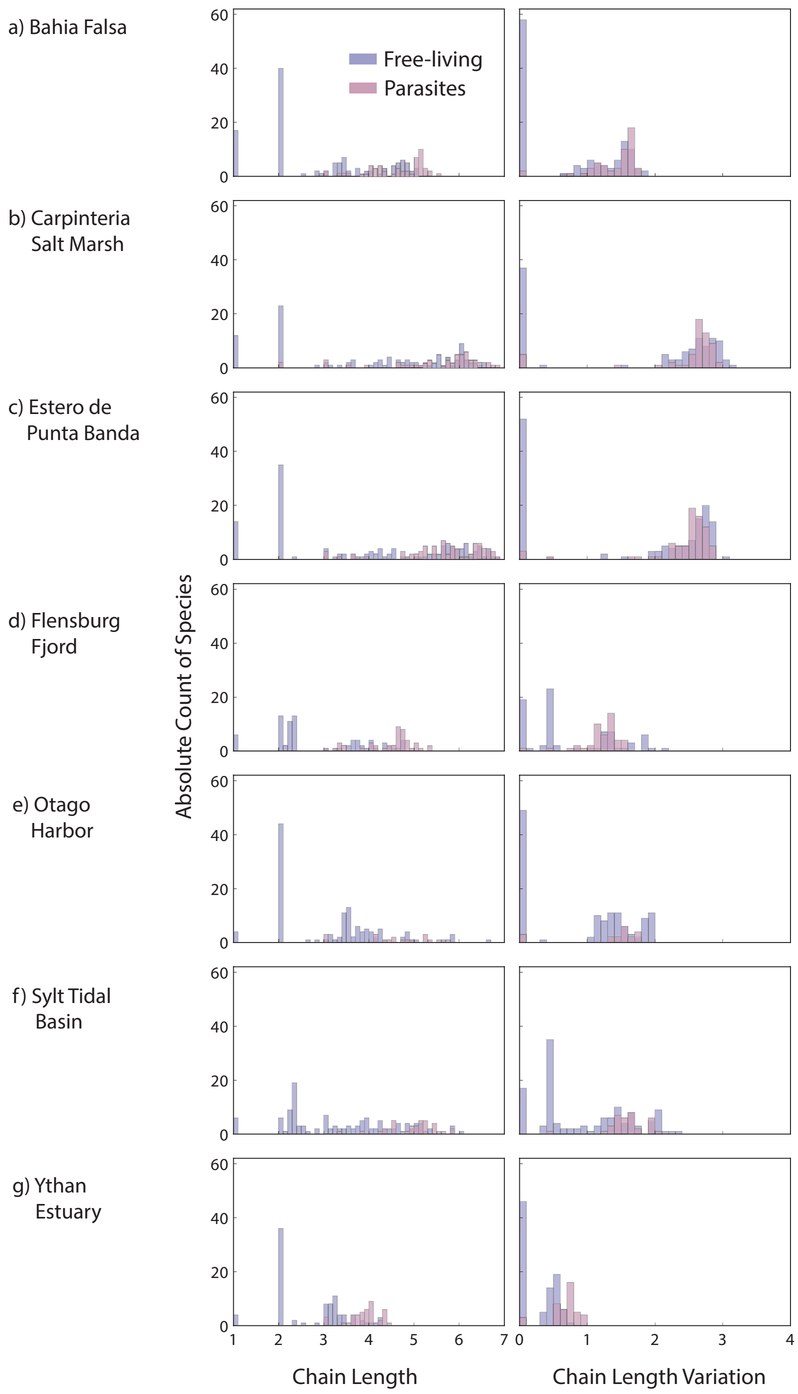

Figure AA: **Mean and standard deviation of chain length for free-living and parasite species separately.** Distribution of mean (left) and standard deviation (right) of species chain lengths for food webs consisting of free-living and parasite species. Free-living and parasite species distinguished by color shades as indicated in legend. **a—g** denote the seven food webs Bahia Falsa, Carpinteria Salt Marsh, Estero de Punta Banda, Flensburg Flord, Otago Harbor, Sylt Tidal Basin, and Ythan Estuary, respectively.

Acknowledgments

The authors acknowledge financial support by the Danish National Research Foundation through the Center for Models of Life.

Conflict of Interest Statement

The authors have no conflict of interest to declare.

References

Bascompte, J., Melián, C. J., and Sala, E. (2005). Interaction strength combinations and the overfishing of a marine food web. *Proceedings of the National Academy of Sciences of the United States of America*, 102(15):5443–5447.

Brose, U., Williams, R. J., and Martinez, N. D. (2006). Allometric scaling enhances stability in complex food webs. *Ecology Letters*, 9(11):1228–1236.

Dunne, J. A., Lafferty, K. D., Dobson, A. P., Hechinger, R. F., Kuris, A. M., Martinez, N. D., , et al. (2013). Parasites affect food web structure primarily through increased diversity and complexity. *PLoS biology*, 11(6):e1001579.

Fagan, W. F. (1997). Omnivory as a stabilizing feature of natural communities. *The American Naturalist*, 150(5):554–567.

Gaedke, U., Straile, D., and Pahl-Wostl, C. (1996). Trophic structure and carbon flow dynamics in the pelagic community of a large lake. In *Food webs*, pages 60–71. Springer.

Goh, B. S. (1977). Global stability in many-species systems. *American Naturalist*, pages 135–143.

Gross, T., Rudolf, L., Levin, S. A., and Dieckmann, U. (2009). Generalized models reveal stabilizing factors in food webs. *Science*, 325(5941):747–750.

Haerter, J. O. and Sneppen, K. (2012). Spatial structure and lamarckian adaptation explain extreme genetic diversity at crispr locus. *mBio*, 3:4.

Heath, M. R., Speirs, D. C., and Steele, J. H. (2014). Understanding patterns and processes in models of trophic cascades. *Ecology letters*, 17(1):101–114.

Hechinger, R. F., Lafferty, K. D., McLaughlin, J. P., Fredensborg, B. L., Huspeni, T. C., Lorda, J., et al. (2011). Food webs including parasites, biomass, body sizes, and life stages for three California/Baja California estuaries: Ecological archives e092-066. *Ecology*, 92(3):791–791.

Hofbauer, J. and Sigmund, K. (1988). *The Theory of Evolution and Dynamical Systems.* Cambridge University Press, pg 59ff.

Huxham, M., Beaney, S., and Raffaelli, D. (1996). Do parasites reduce the chances of triangulation in a real food web? *Oikos*, 76:284–300.

Johnson, P. T., Dobson, A., Lafferty, K. D., Marcogliese, D. J., Memmott, J., Orlofske, S. A., et al. (2010). When parasites become prey: ecological and epidemiological significance of eating parasites. *Trends in Ecology & Evolution*, 25(6):362–371.

Lafferty, K. D., Allesina, S., Arim, M., Briggs, C. J., De Leo, G., Dobson, A. P., et al. (2008). Parasites in food webs: the ultimate missing links. *Ecology letters*, 11(6):533–546.

Lafferty, K. D., Dobson, A. P., and Kuris, A. M. (2006). Parasites dominate food web links. *Proceedings of the National Academy of Sciences*, 103(30):11211–11216.

Lafferty, K. D. and Morris, A. K. (1996). Altered behavior of parasitized killifish increases susceptibility to predation by bird final hosts. *Ecology*, 77(5):1390–1397.

Lovász, L. and Plummer, M. D. (1986). Matching theory. *New York*.

MacArthur, R. H. (1967). *The theory of island biogeography*, volume 1. Princeton University Press.

McCann, K. S. (2000). The diversity–stability debate. *Nature*, 405(6783):228–233.

McPeek, M. A. (1996). Trade-offs, food web structure, and the coexistence of habitat specialists and generalists. *American Naturalist*, pages S124–S138.

Montoya, J. M., Pimm, S. L., and Solé, R. V. (2006). Ecological networks and their fragility. *Nature*, 442(7100):259–264.

Mordecai, E. A. (2011). Pathogen impacts on plant communities: unifying theory, concepts, and empirical work. *Ecological Monographs*, 81(3):429–441.

Mougi, A. and Kondoh, M. (2012). Diversity of interaction types and ecological community stability. *Science*, 337(6092):349–351.

Mouritsen, K. N., Poulin, R., McLaughlin, J. P., and Thieltges, D. W. (2011). Food web including metazoan parasites for an intertidal ecosystem in New Zealand: Ecological Archives E092-173. *Ecology*, 92(10):2006–2006.

Otto, S. P. and Day, T. (2007). *A biologist's guide to mathematical modeling in ecology and evolution*, volume 13. Princeton University Press.

Pauly, D. (1996). Fishing down marine food webs. *Science*, 273:1706.

Smith, J. M. and Slatkin, M. (1973). The stability of predator-prey systems. *Ecology*, 54(2):384–391.

Tao, T. and Vu, V. (2006). On random± 1 matrices: singularity and determinant. *Random Structures & Algorithms*, 28(1):1–23.

Thieltges, D. W., Reise, K., Mouritsen, K. N., McLaughlin, J. P., and Poulin, R. (2011). Food web including metazoan parasites for a tidal basin in Germany and Denmark: Ecological Archives E092-172. *Ecology*, 92(10):2005–2005.

Thingstad, T. F. (2000). Elements of a theory for the mechanisms controlling abundance, diversity, and biogeochemical role of lytic bacterial viruses in aquatic systems. *Limnology and Oceanography*, 45(6):1320–1328.

Thompson, R. M., Hemberg, M., Starzomski, B. M., and Shurin, J. B. (2007). Trophic levels and trophic tangles: the prevalence of omnivory in real food webs. *Ecology*, 88(3):612–617.

Tilman, D. (1990). Constraints and tradeoffs: toward a predictive theory of competition and succession. *Oikos*, pages 3–15.

Tilman, D. (1994). Competition and biodiversity in spatially structured habitats. *Ecology*, 75(1):2–16.

Tilman, D. (2000). Causes, consequences and ethics of biodiversity. *Nature*, 405(6783):208–211.

Tilman, D. (2011). Diversification, biotic interchange, and the universal trade-off hypothesis. *The American Naturalist*, 178(3):355–371.

Williams, R. J. and Martinez, N. D. (2004). Limits to trophic levels and omnivory in complex food webs: theory and data. *The American Naturalist*, 163(3):458–468.

Wilson, E. E. and Wolkovich, E. M. (2011). Scavenging: how carnivores and carrion structure communities. *Trends in ecology & evolution*, 26(3):129–135.

Zander, C. D., Josten, N., Detloff, K. C., Poulin, R., McLaughlin, J. P., and Thieltges, D. W. (2011). Food web including metazoan parasites for a brackish shallow water ecosystem in Germany and Denmark: Ecological Archives E092-174. *Ecology*, 92(10):2007–2007.
